# Supplementary material for: Matrix prior for data transfer between single cell data types in latent Dirichlet allocation
Source: PLoS Comput Biol. 2023 May 5;19(5):e1011049. doi: 10.1371/journal.pcbi.1011049 (PMC10191269; doi:10.1371/journal.pcbi.1011049)
Supplement: S1 Table — (PDF) [file pcbi.1011049.s001.pdf]

Variable	Definition
N	The number of reads in a cell
T	The number of topics
V	The number of peaks or genes in the vocabulary
U	The number of cells
$\boldsymbol{w}$	A vector of genes or peaks in a cell
$\boldsymbol{\theta}$	A vector $(\theta_1, \ldots, \theta_T)$; the topic distribution of a given cell
$\hat{\boldsymbol{\theta}}$	Inferred $\boldsymbol{\theta}$
$\boldsymbol{\phi}$	A matrix such that ϕ_{tw} is the probability of observing a peak or gene w for a topic t
$\hat{\boldsymbol{\phi}}$	Inferred $\boldsymbol{\phi}$
$\boldsymbol{\alpha}$	A basis vector of length T that parameterizes the Dirichlet prior distribution over the cell-topic matrix.
$\boldsymbol{\beta}$	A basis vector of length V that parameterizes the Dirichlet prior distribution over the topic-peak/gene matrix.
c_α	The concentration parameter for $\boldsymbol{\alpha}$
c_β	The concentration parameter for $\boldsymbol{\beta}$
$\boldsymbol{z}$	A vector of topic assignments corresponding to $\boldsymbol{w}$
ξ	The average number of peaks or genes in a cell
$\boldsymbol{B}$	A $T \times V$ matrix that parameterizes the matrix prior for the topic-peak/gene matrix.
c_B	A concentration parameter for $\boldsymbol{B}$
$\hat{\boldsymbol{\phi}}_{ref}$	The output inferred $\boldsymbol{\phi}$ of an LDA analysis on the reference dataset.
