# Supplementary material for: Matrix prior for data transfer between single cell data types in latent Dirichlet allocation
Source: PLoS Comput Biol. 2023 May 5;19(5):e1011049. doi: 10.1371/journal.pcbi.1011049 (PMC10191269; doi:10.1371/journal.pcbi.1011049)
Supplement: S1 Note — We investigated the use of the scRNA-seq matrix prior. (PDF) [file pcbi.1011049.s002.pdf]

Supplementary note 1: SHARE-seq scRNA-seq matrix prior may have limited use

We investigated the feasibility of using scRNA-seq data to construct a matrix prior for scATAC-seq analysis. This required that we map the scRNA-seq data and the scATAC-seq data onto a shared feature axis. To accomplish this mapping, translated the scATAC-seq data from a vocabulary of peaks to one of genes by simply counting the number of scATAC-seq cut sites overlapping each gene and its promoter (see Methods). We then proceeded with a similar analysis to Sections 4.2 and 4.3. To evaluate the performance of the matrix prior, we leveraged the fact that the scATAC-seq data and scRNA-seq data were generated from the same set of cells, and compared the inferred cell-topic and topic-gene matrices from the matrix prior LDA that was trained on the target scATAC-seq dataset to the output matrices generated by a joint model on the full scRNA-seq dataset.

We found that using the scRNA-seq data to construct the matrix prior did not consistently improve inference of the cell-topic and topic-gene matrices, although we saw some improvement at moderate values of c_B. For the cell-topic matrices, we found that as the concentration parameter c_B increased beyond 4,000, the Pearson correlation to the joint model output tended to decrease. Similarly, Spearman correlation and MSE both got worse as c_B increased beyond 4,000. For the topic-gene matrices, however, Spearman correlation improved as c_B increased (Figs 2, S12). When plotting the raw values of the cell-topic matrix, we observed more points along the x- and y-axes. The reference model and joint model assigned low probability topics to different cells more frequently as c_B increased (S14 Fig). For the topic-gene matrix, the diagonal had increased density, meaning that the two analyses agreed more as c_B increased (S15 Fig)

We note that the read counts per gene and per cell in scRNA-seq was only moderately correlated with the number of cut sites summed over the gene body in the scATAC-seq data, with a Pearson correlation of 0.661 for the signal per cell, and 0.765 for the signal per gene (S6 Fig). This makes sense because, although chromatin accessibility and gene expression are highly related biological phenomena, the information encoded by scATAC-seq and scRNA-seq count data is not the same. This could be one reason why the matrix prior only improves results compared to the uniform prior at moderate values of c_B. If a prior derived from a different data modality is given too much weight, then the discrepancies between the modalities are more likely to lead to a poor model fit.
