# Supplementary material for: Matrix prior for data transfer between single cell data types in latent Dirichlet allocation
Source: PLoS Comput Biol. 2023 May 5;19(5):e1011049. doi: 10.1371/journal.pcbi.1011049 (PMC10191269; doi:10.1371/journal.pcbi.1011049)
Supplement: S2 Note — We evaluated silhouette values in the C. elegans dataset. (PDF) [file pcbi.1011049.s003.pdf]

Supplementary Note 2: *C. elegans* silhouette values did not improve through use of the prior

The *C. elegans* data was previously labeled with a cell type based on marker genes through a clustering method that took into account all scATAC-seq peaks using all the cells together. We hypothesized that we would be better able to recover these cell type labels in the target dataset by incorporating information from the matrix prior. To this end, we analyzed the target dataset using both the matrix prior derived from a reference subset of cells and the uniform prior, and then evaluated how well the clusters in the cell-topic output agreed with the published cell type labels.

We used UMAP [McInnes et al., 2018] to reduce the 15-dimensional topic space to a two-dimensional representation and then colored each cell according to its cell type label. We compared these UMAP plots of the results of our LDA analysis with the matrix prior and with the uniform prior, as well as with different weights of c_B (S17a Fig). The UMAP plots show that all of the LDA models produce reasonable agreement with the cell type labels, although we note that quantifying the cell type discrimination in each LDA output with the silhouette score shows a slight increase in the mean silhouette value, from 0.216 with the uniform prior to 0.220 with the matrix prior and $c_B = 4000$ (Figs S16, S17b).

Despite the similarity of the UMAP plots, we noted that at $c_B = 4000$ the neurons appeared to split into two clusters, and the average silhouette score for the neurons decreased from 0.254 with the uniform prior to 0.109 with the matrix prior and $c_B = 4000$. This observation suggested the hypothesis that the use of the prior might help to resolve fine grained cell types, so we repeated our cell type label analysis specifically on the neurons, this time using the published neuron subtype labels (S18a Fig). The UMAP plots show that as c_B increased the cells were clustered more tightly together, however there was little change in how well the cell types were separated compared to the LDA with the uniform prior. In addition, the mean silhouette scores for the neuron subtypes (S18b Fig), did not increase as the value of c_B increased. Overall, these analyses do not suggest that the neuron subtypes were further resolved by the use of the matrix prior.
