# Supplementary figures and images for: Matrix prior for data transfer between single cell data types in latent Dirichlet allocation
Source: PLoS Comput Biol. 2023 May 5;19(5):e1011049. doi: 10.1371/journal.pcbi.1011049 (PMC10191269; doi:10.1371/journal.pcbi.1011049)

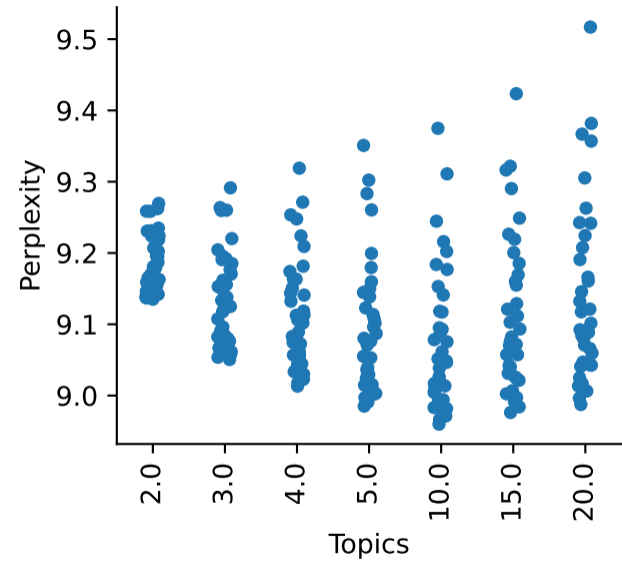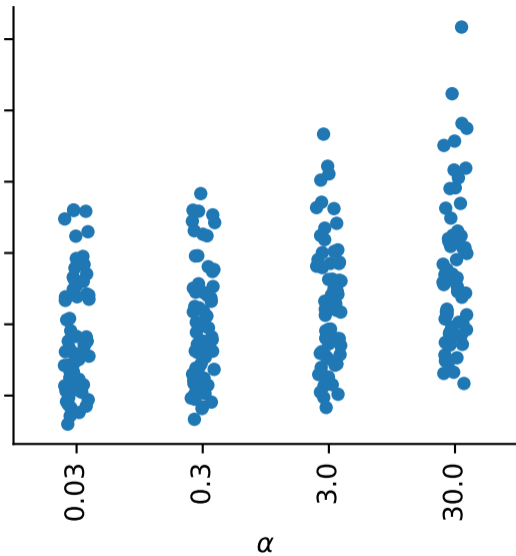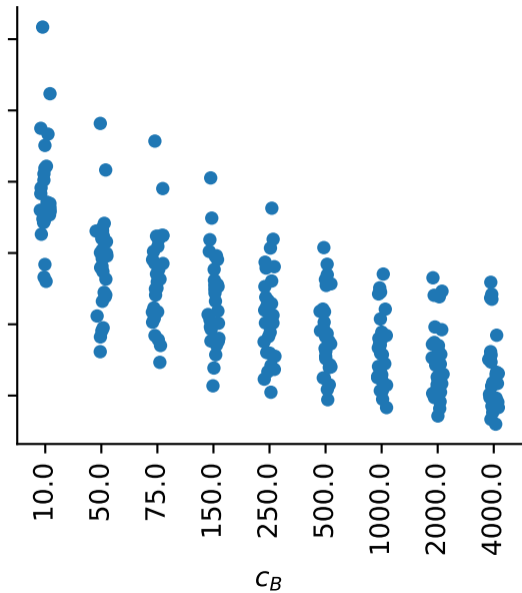

Supplement: S1 Fig — Hyperparameter search for C. elegans data was used to optimize the parameters. Each point is the average of 10 folds of the perplexity value of the test set. The x-axis is the value of the hyperparameter, and the y-axis is the perplexity value. (PDF) [file pcbi.1011049.s004.pdf]

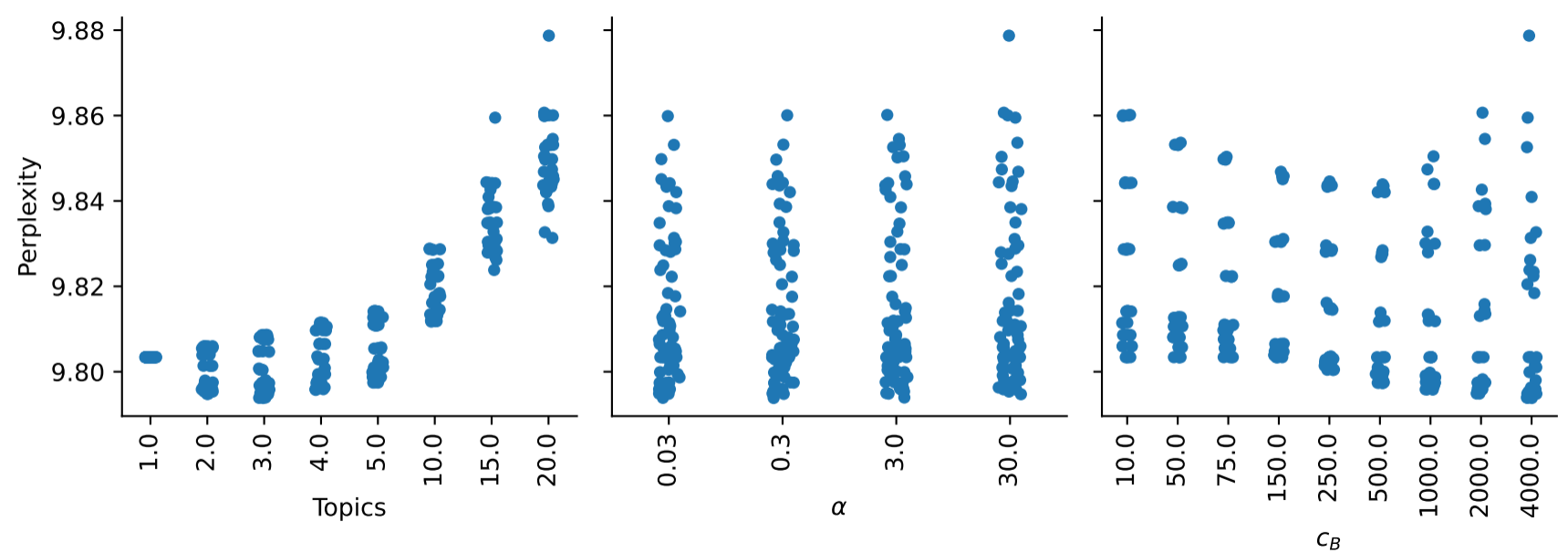

Supplement: S2 Fig — Hyperparameter search for SHARE-seq data with peaks was used to optimize the parameters. Each point is the average of 10 folds of the perplexity value of the test set. The x-axis is the value of the hyperparameter, and the y-axis is the perplexity value. (PDF) [file pcbi.1011049.s005.pdf]

# UMAP Plots with $\alpha=0.030$ , Beta=4000

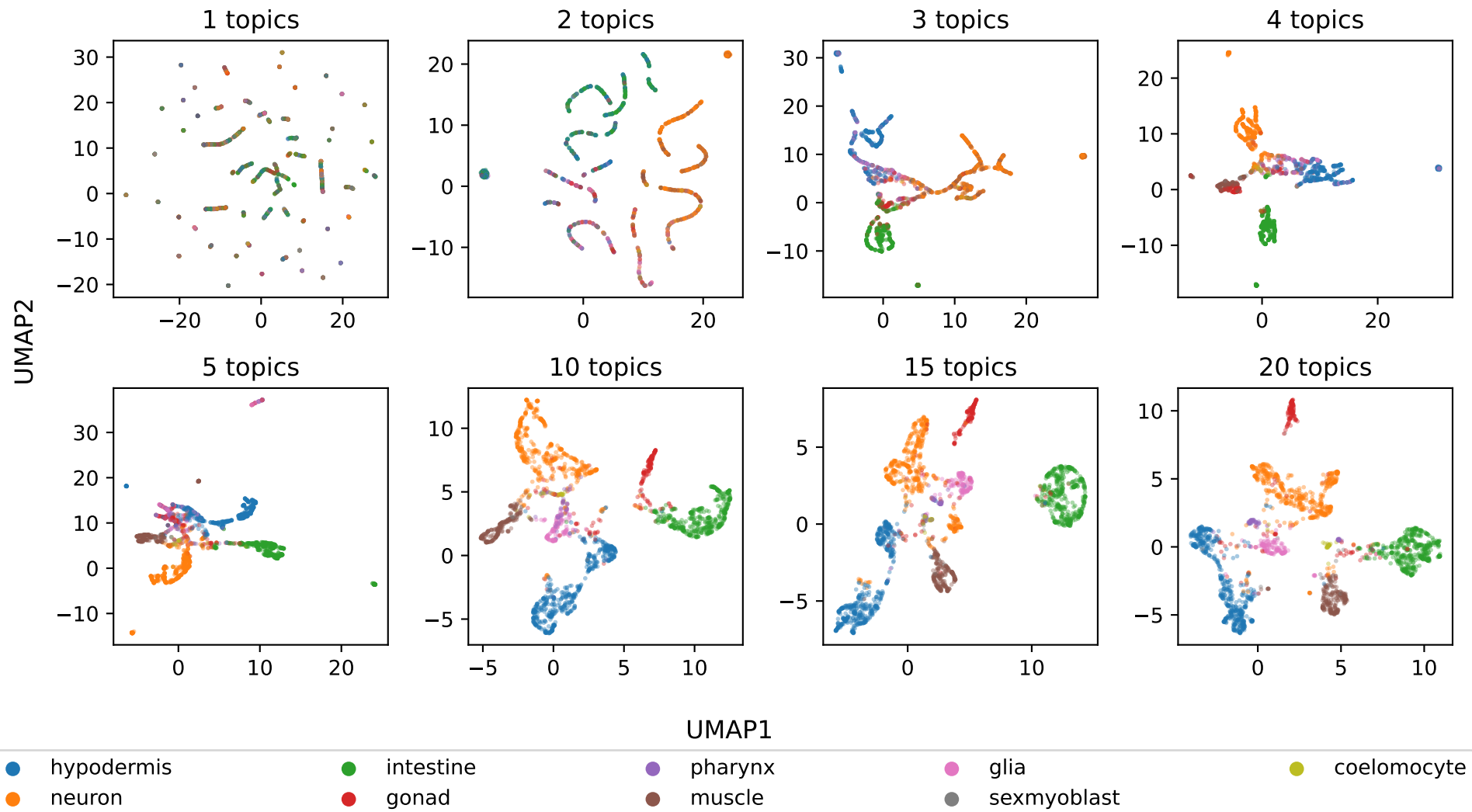

Supplement: S3 Fig — UMAP embeddings for different numbers of topics are shown to provide intuition on the effect of the number of topics. Embeddings were made for cell-topic matrices from matrix prior LDA on C. elegans scATAC-seq data using a fixed value of cB = 4, 000 and 13,734 peaks, while varying the number of topics. (PDF) [file pcbi.1011049.s006.pdf]

UMAP Plots with  $\alpha=0.030$ ,  $c_\beta=4000$

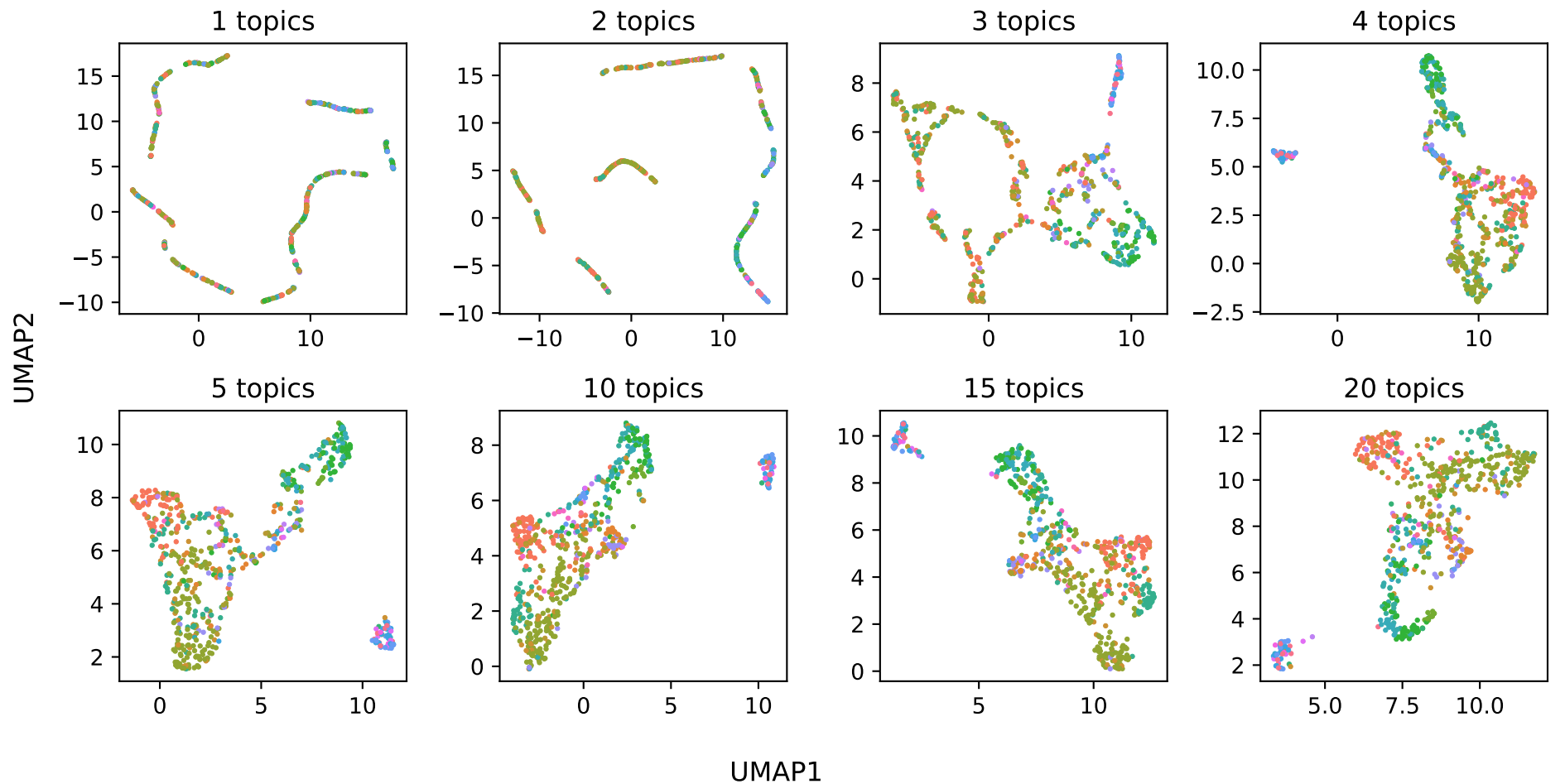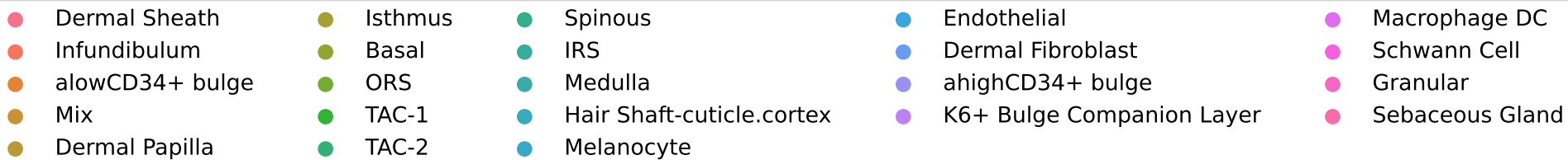

Supplement: S4 Fig — UMAP embeddings for different numbers of topics are shown to provide intuition on the effect of the number of topics. Plots were made with 630 target cells from the mouse skin SHARE-seq peak data subsetted to 7,000 cells and 20,000 most variable peaks. LDA was run with cβ = 4, 000 using the uniform prior, while varying the number of topics. (PDF) [file pcbi.1011049.s007.pdf]

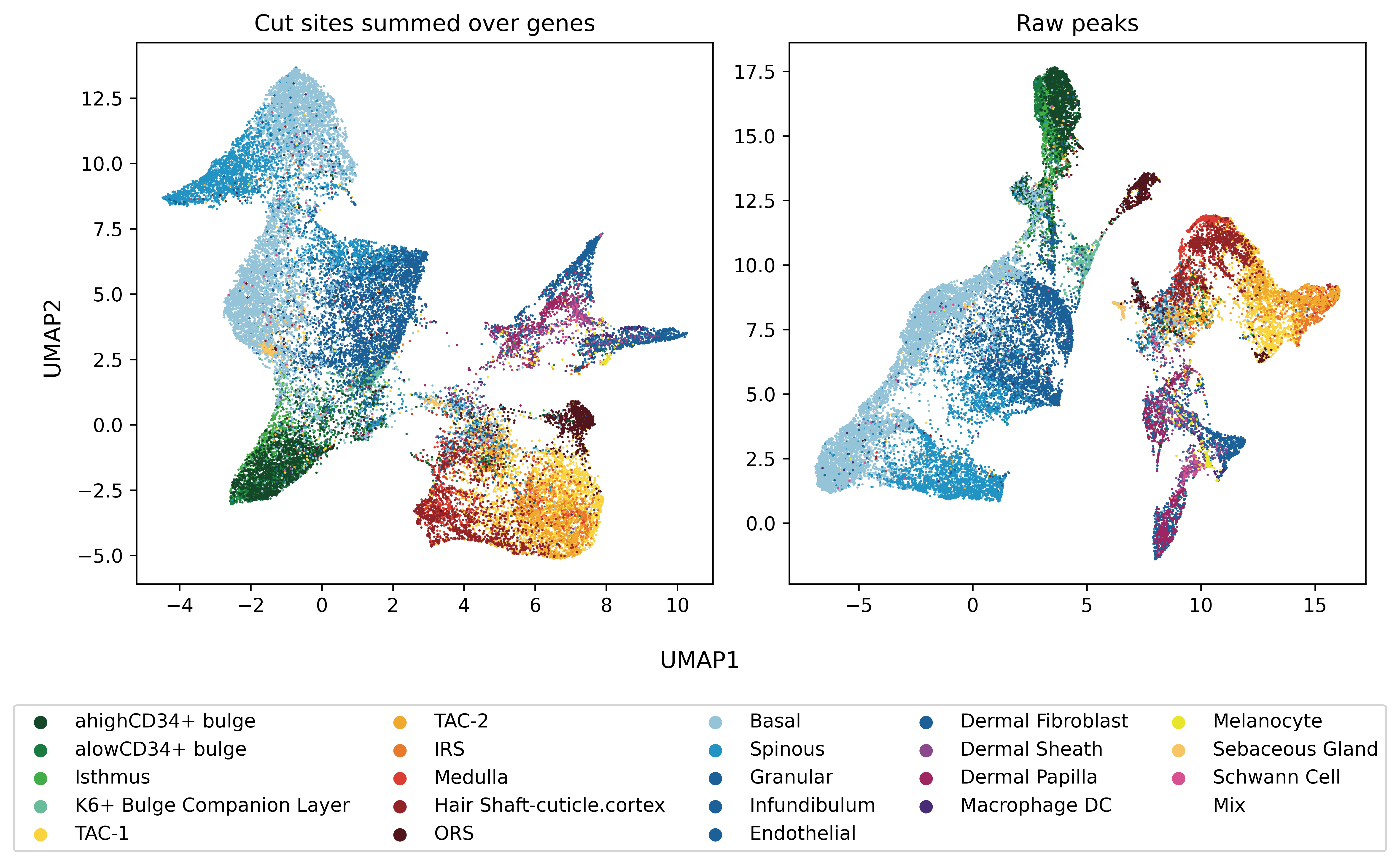

Supplement: S5 Fig — UMAP was applied to the cell-topic matrices output from LDA joint model to qualitatively compare cut sites summed over genes versus peaks. LDA was run with 15 topics, cα = 3, and cβ = 4000. On the left, the raw data fed into LDA are the cut sites summed over the 22,813 genes, as described in Section 2.2.4. On the right, the data fed into LDA are the 344,592 raw peaks. (PNG) [file pcbi.1011049.s008.png]

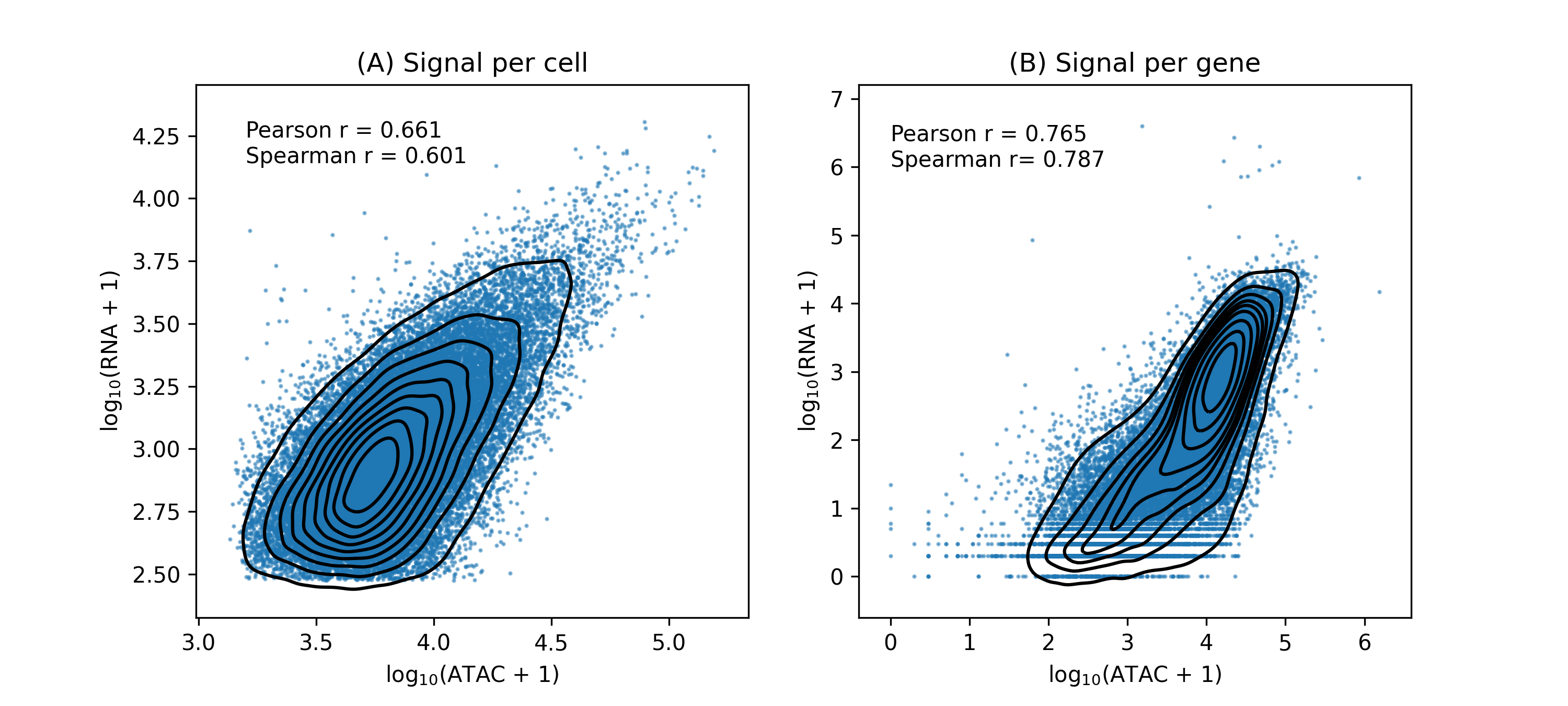

Supplement: S6 Fig — RNA reads and scATAC-seq cut sites summed over gene bodies are compared to determine the shared information between the data modalities. On the left, the signal per cell is the log plus one of the total number of counts for each cell (i.e. summing across all the genes in a cell). On the right, the signal per gene is the log plus one of the total number of counts for each gene (i.e. summing across all the cells for a gene). The Pearson correlation is reported in each plot. A kernel density estimator is overlayed on the data. The x-axis shows the score for the scATAC-seq data, and the y-axis shows the score for the scRNA-seq data. (PNG) [file pcbi.1011049.s009.png]

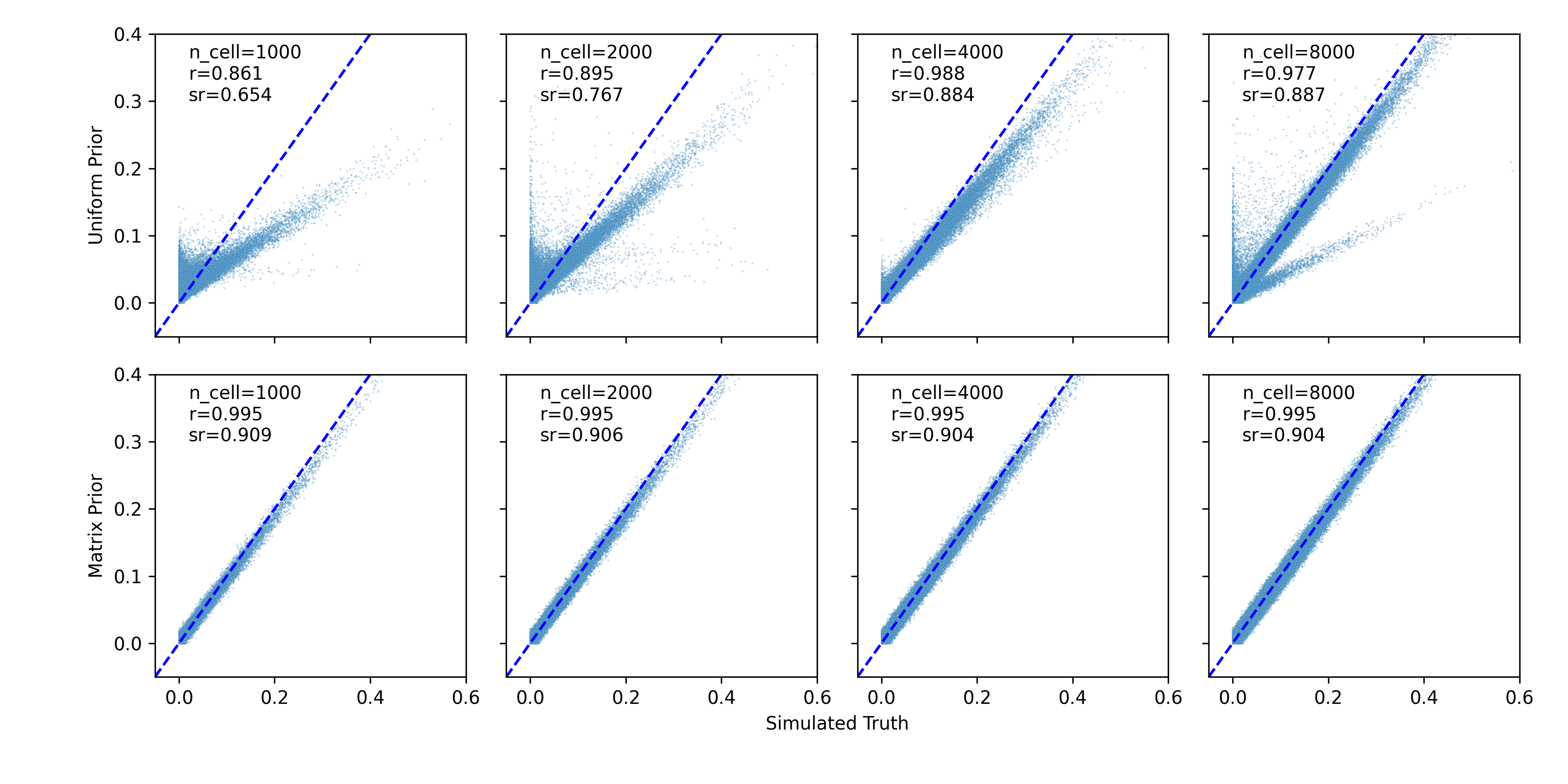

Supplement: S7 Fig — Scatter plots of cell-topic matrix values demonstrate the improvement of the matrix prior over the uniform prior in the true matrix simulation and further show that the performance of the uniform prior approaches that of the matrix prior as the number of cells increases. Plots show simulated true values (x-axis) of the cell-topic matrix against inferred values using LDA (y-axis). Pearson r (r) and Spearman r (sr) are reported for each plot. We compared different numbers of cells in the target dataset (different columns). We compared LDA with a uniform prior (top row) with a matrix prior generated from the true topic-gene matrix (bottom row). The blue dotted line is the line y = x. (PNG) [file pcbi.1011049.s010.png]

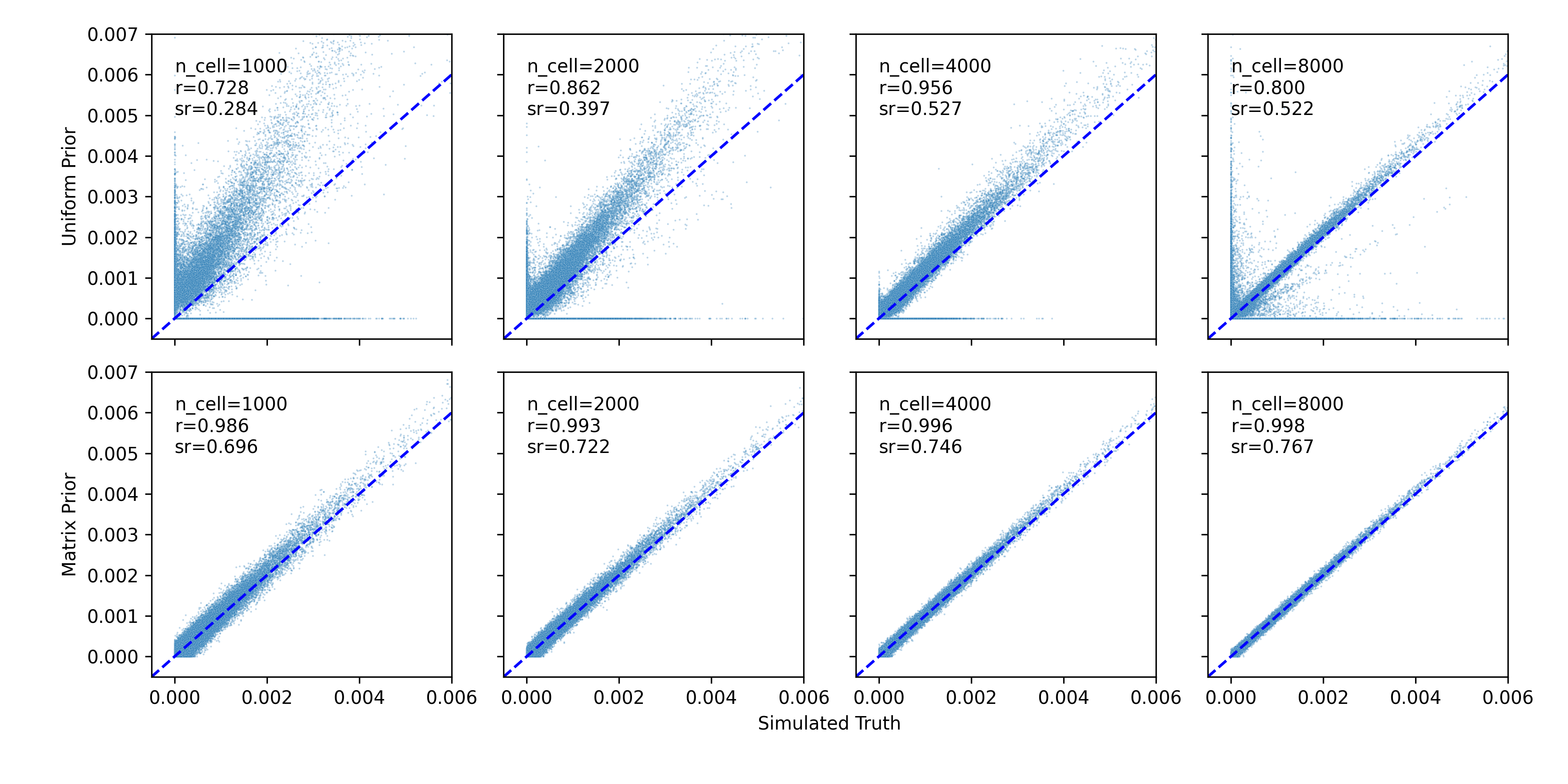

Supplement: S8 Fig — Scatter plots of topic-gene matrix values demonstrate the improvement of the matrix prior over the uniform prior in the true matrix simulation and further show that the performance of the uniform prior approaches that of the matrix prior as the number of cells increases. Plots show simulated true values (x-axis) of the topic-gene matrix against inferred values using LDA (y-axis). Pearson r (r) and Spearman r (sr) are reported for each plot. We compared different numbers of cells in the target dataset (different columns). We compared LDA with a uniform prior (top row) with a matrix prior generated from the true topic-gene matrix (bottom row). The blue dotted line is the line y = x. (PNG) [file pcbi.1011049.s011.png]

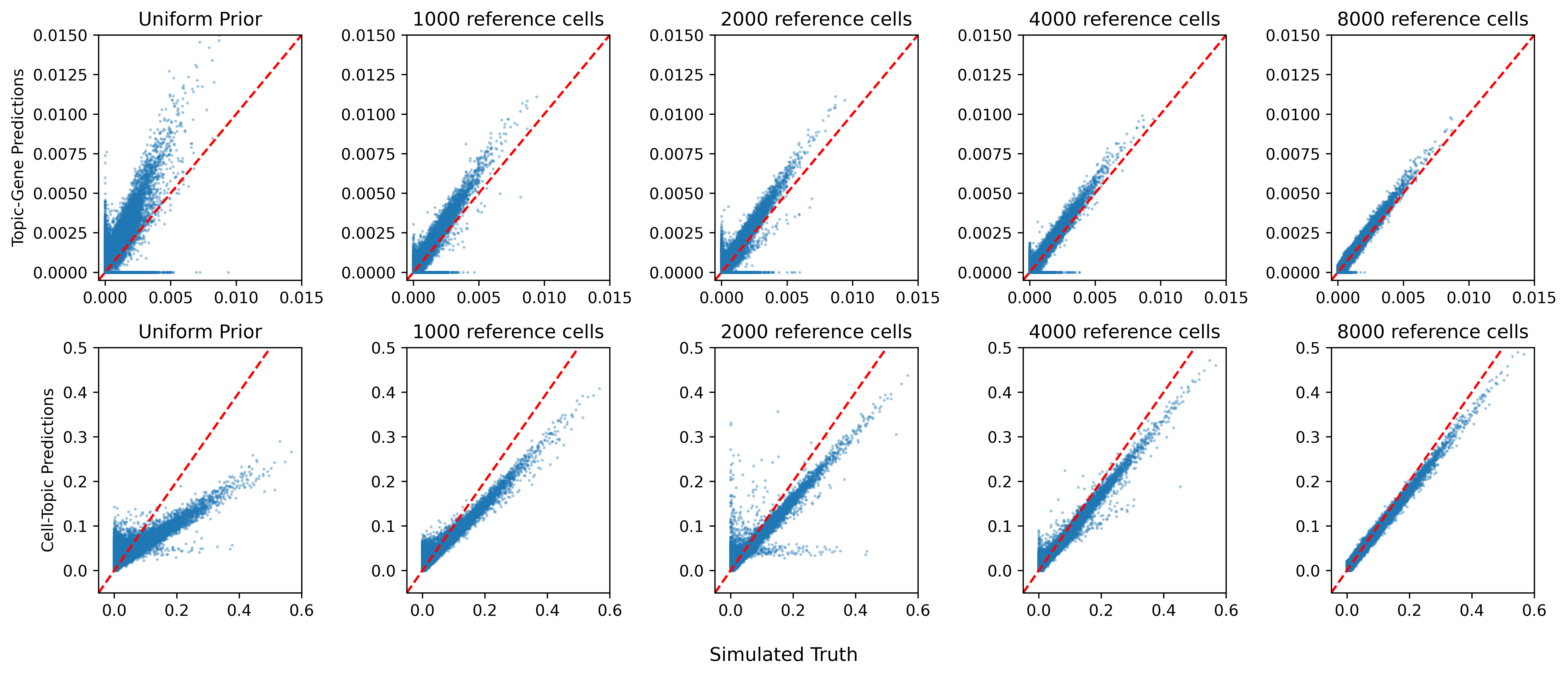

Supplement: S9 Fig — Scatter plots demonstrate the improvement of the matrix prior in both the cell-topic and topic-gene matrices as the number of reference cells increases in the inferred matrix simulation. 1000 simulated cells were analyzed using a uniform prior (left-most column) and a matrix prior. The dotted red line is the y = x line. True simulated values (x-axis) and inferred values (y-axis) are plotted for both the topic-gene matrices (top) cell-topic matrices (bottom). (PNG) [file pcbi.1011049.s012.png]

*C. elegans* Cell-Topic

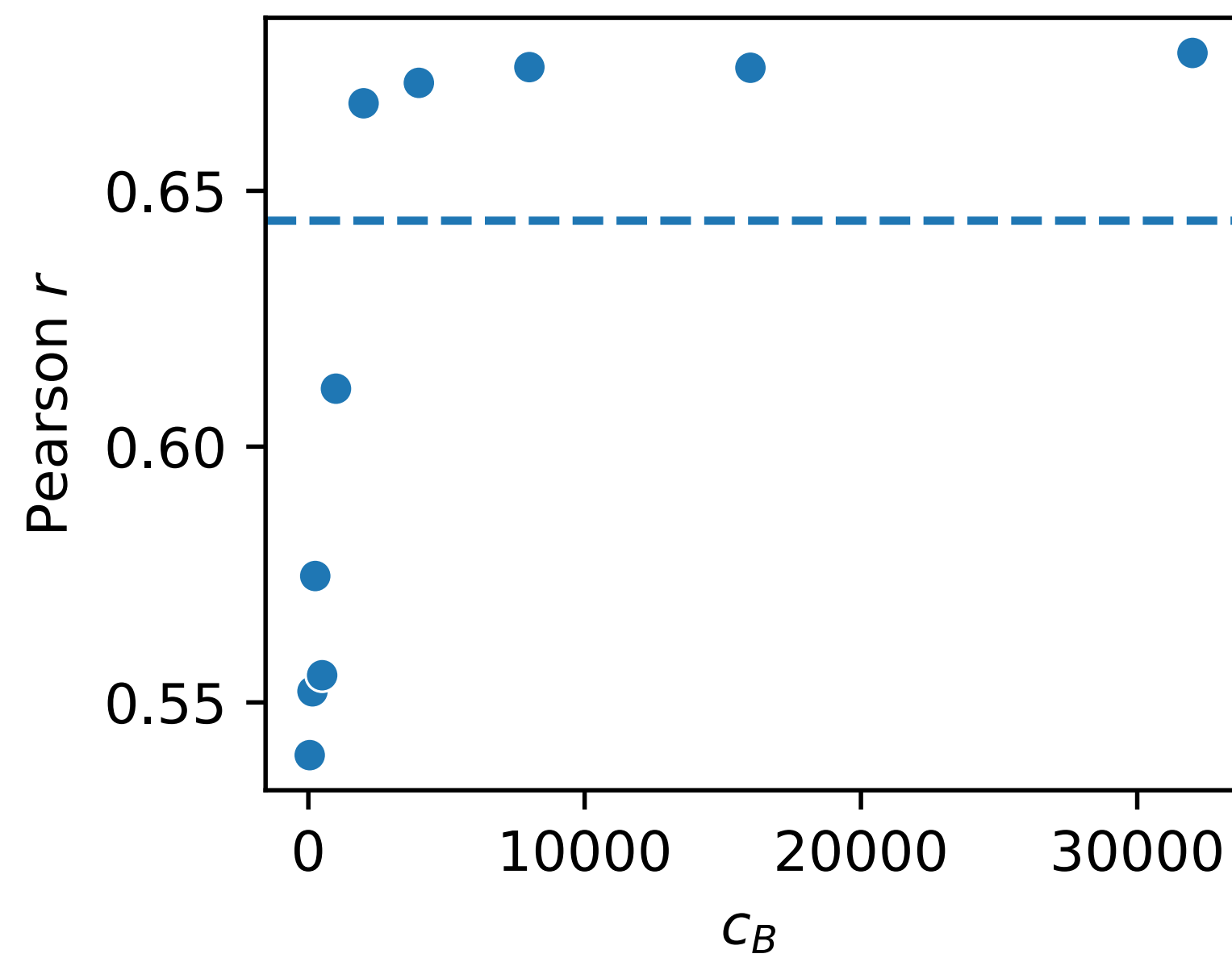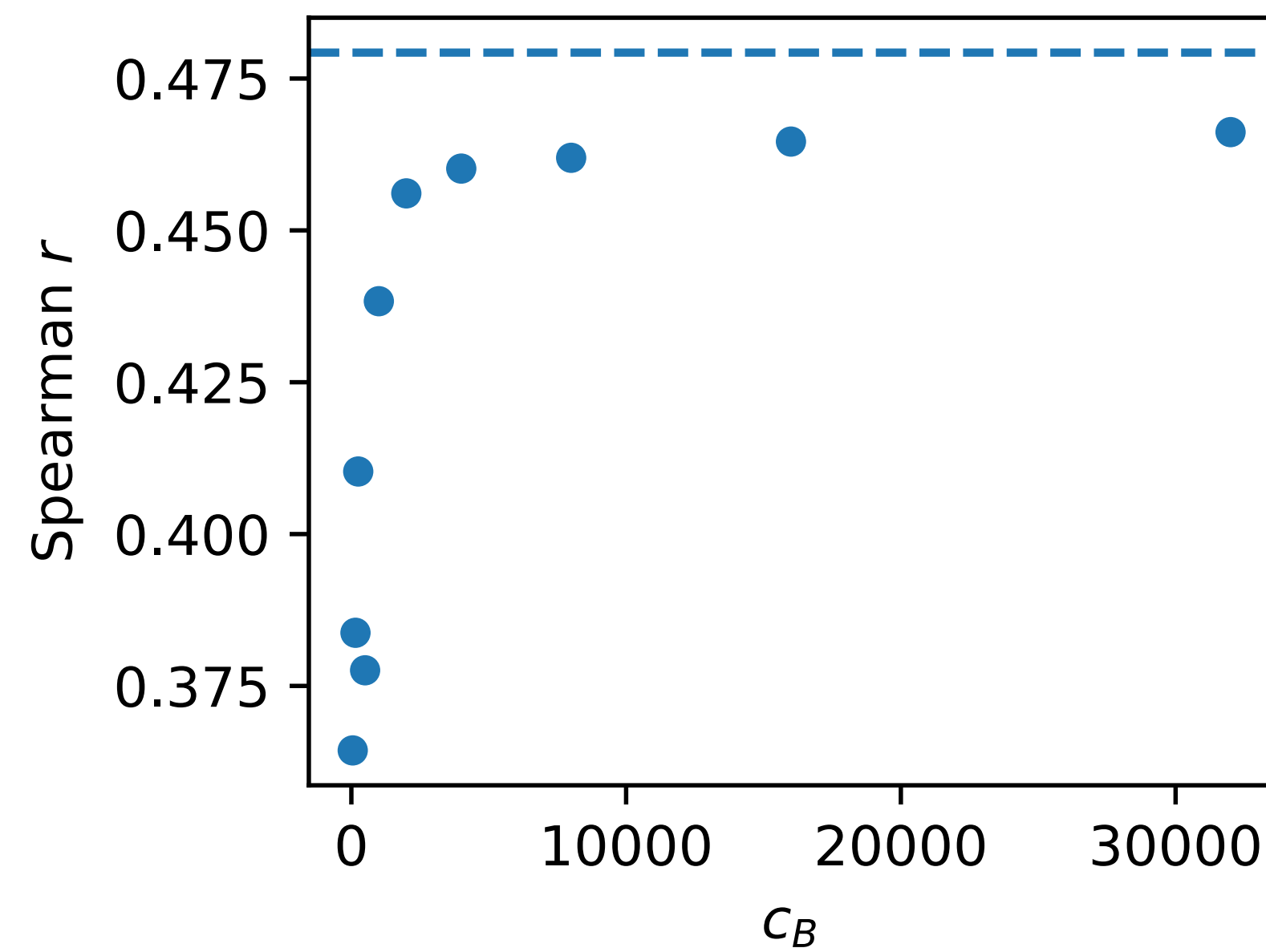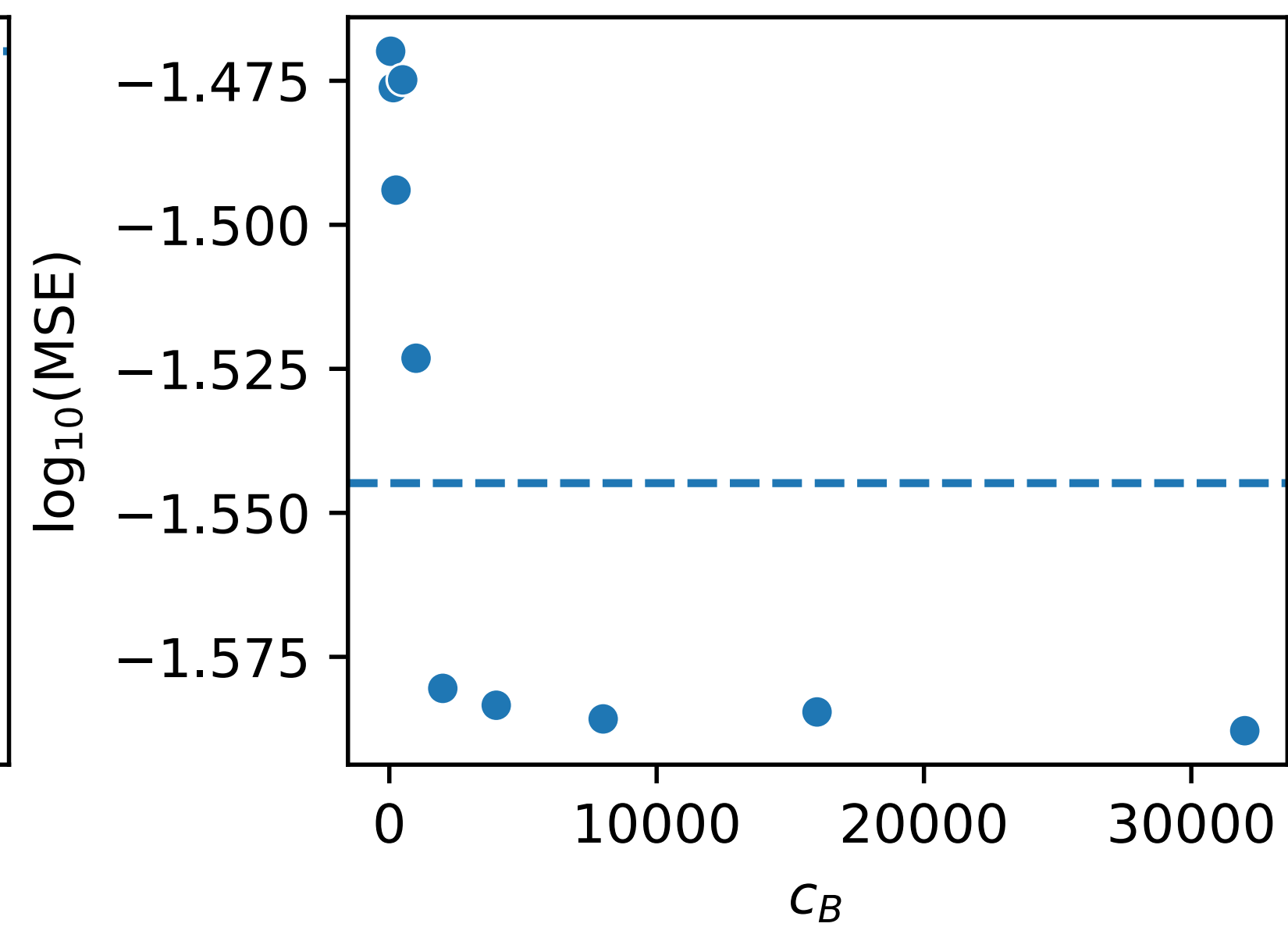

*C. elegans* Topic-Gene

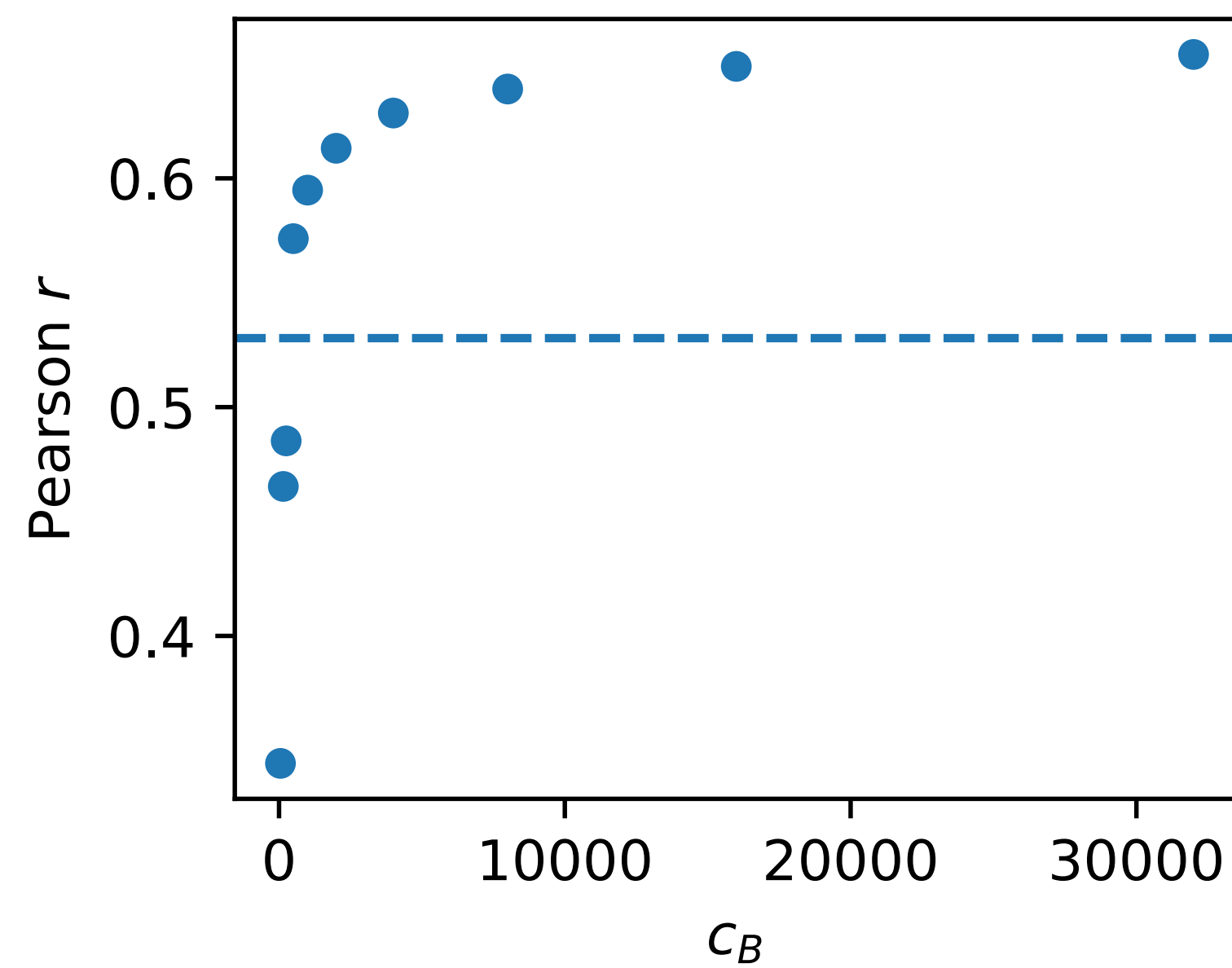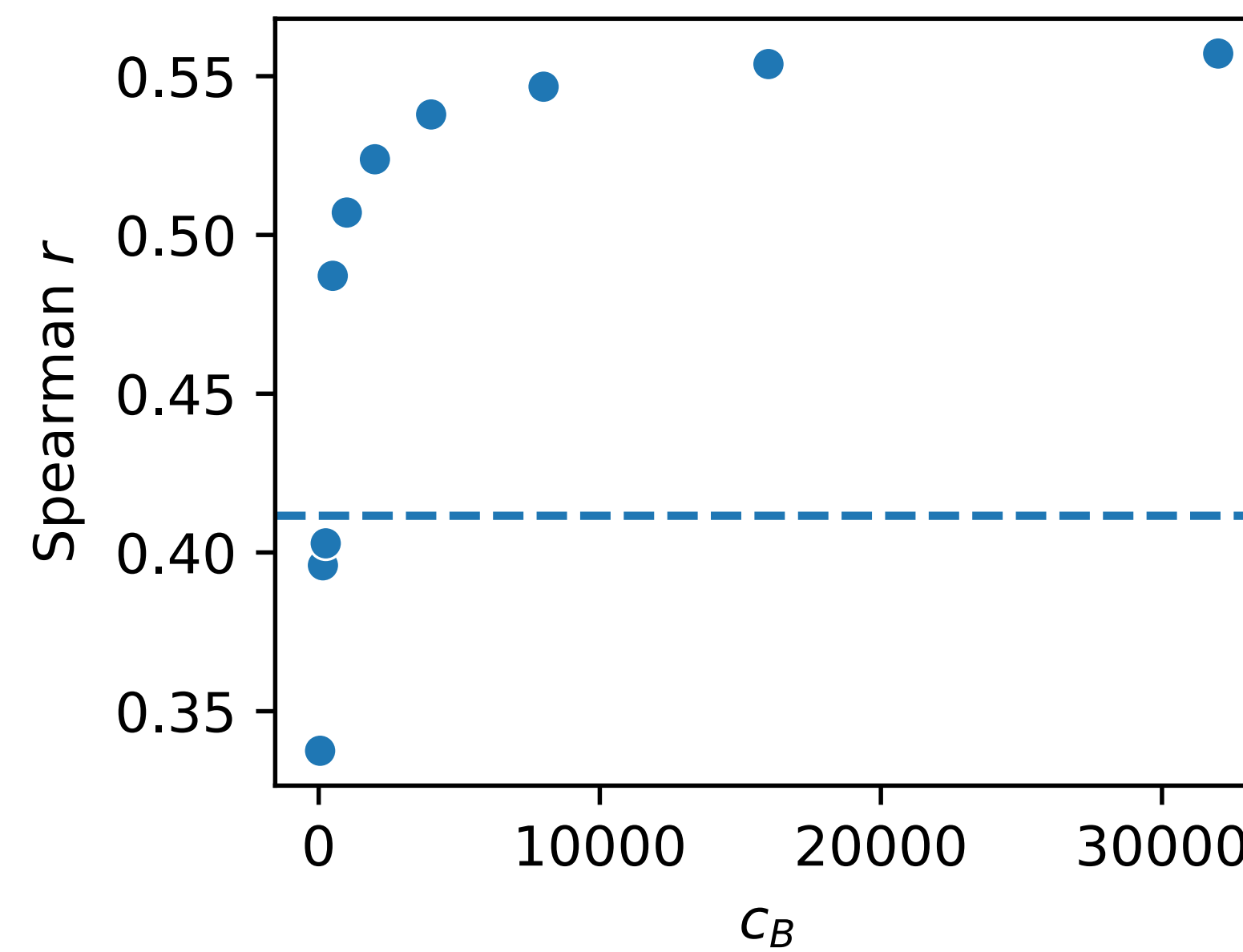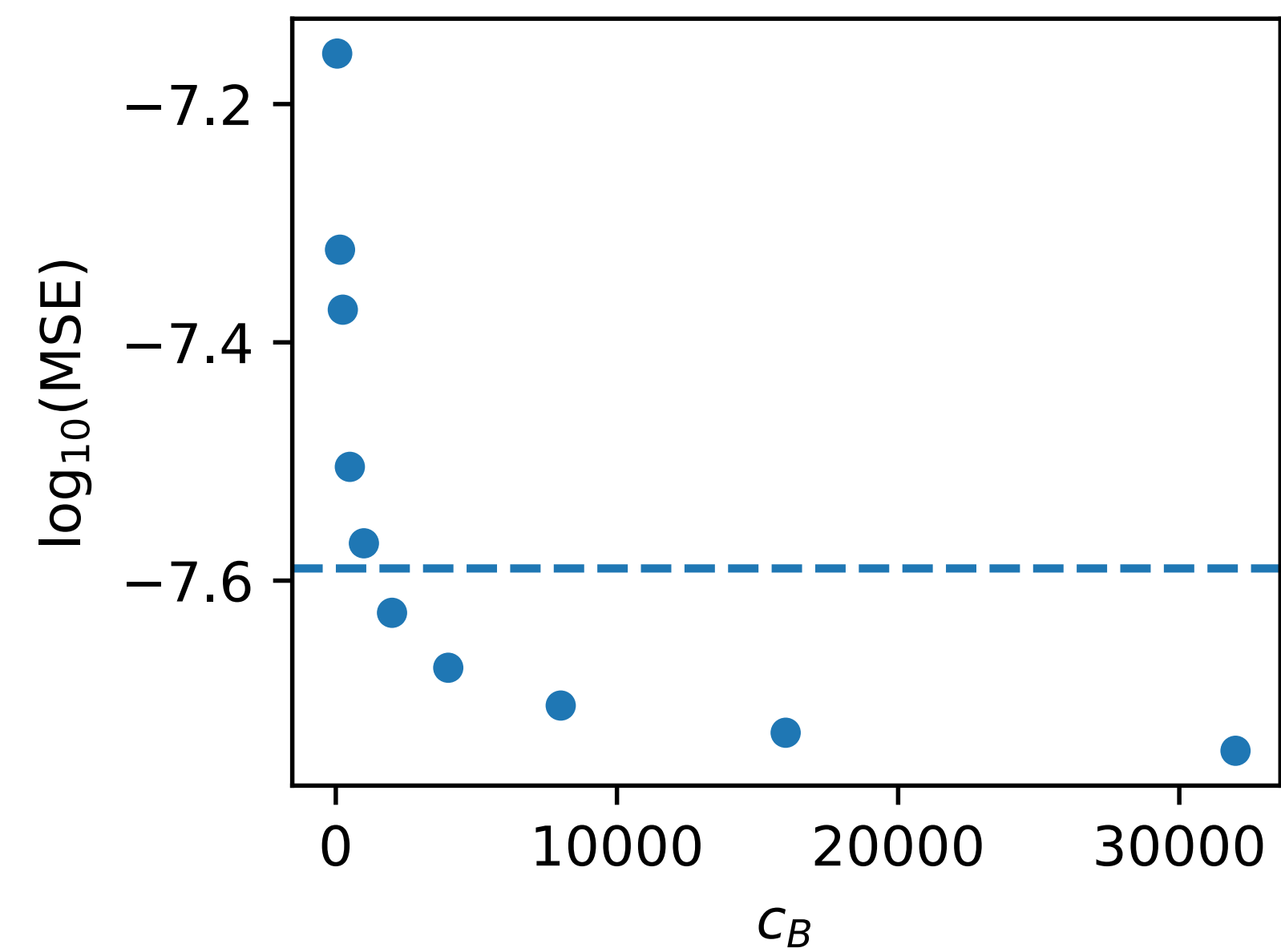

Supplement: S10 Fig — Summary statistics (y-axis) between output matrices of the joint model and LDA with matrix prior, both trained on the C. elegans data, show that as the weight of the prior increases, agreement between the matrix prior and joint model also increases. Each plot shows the matrix prior LDA results (points) for increasing values of cB (x-axis) versus the uniform prior (blue dotted line). The top row of plots shows summary statistics for the cell-topic matrix, and the bottom row of plots shows summary statistics for the topic-gene matrix. (PDF) [file pcbi.1011049.s013.pdf]

Mouse Skin Genes (ATAC) Cell-Topic

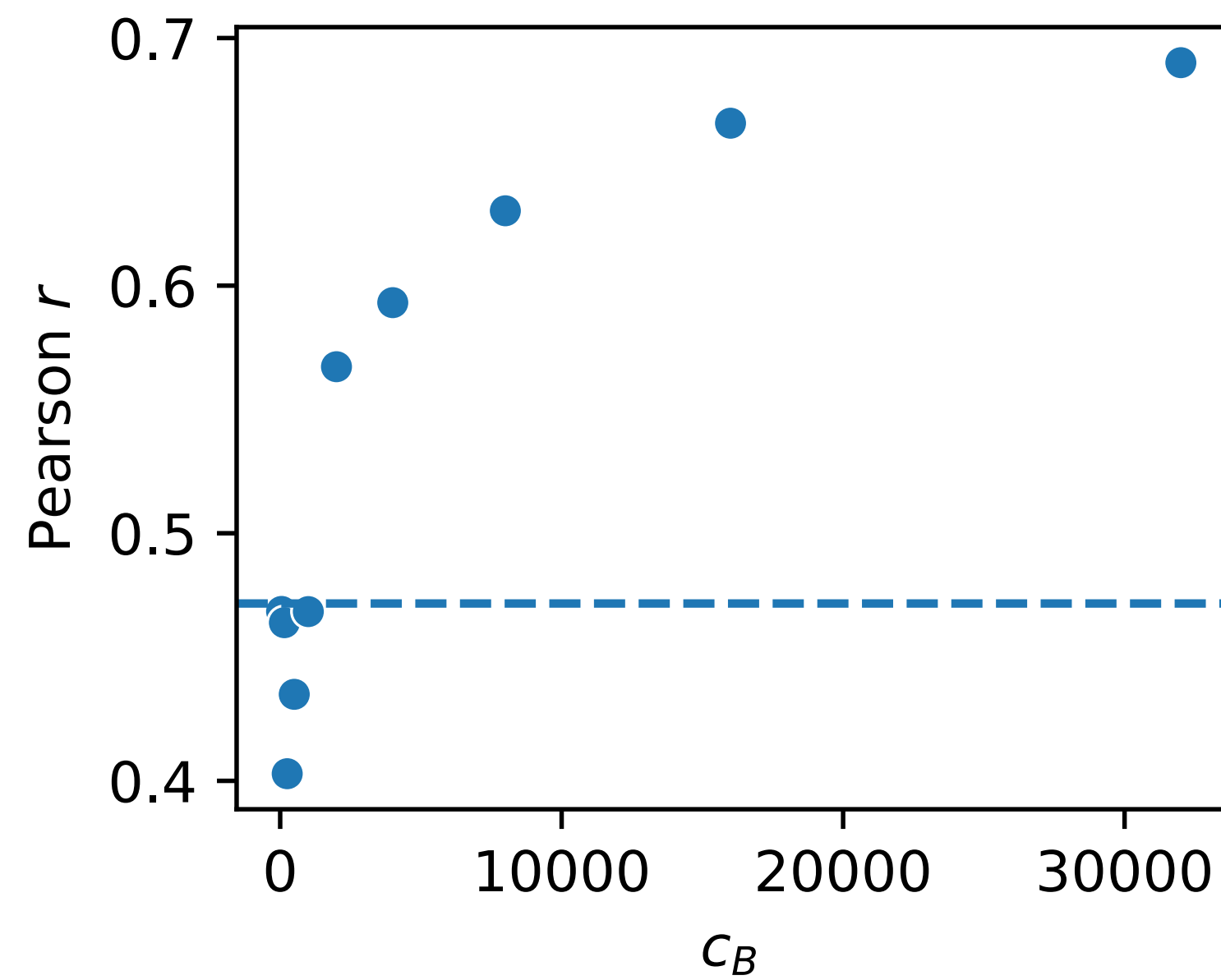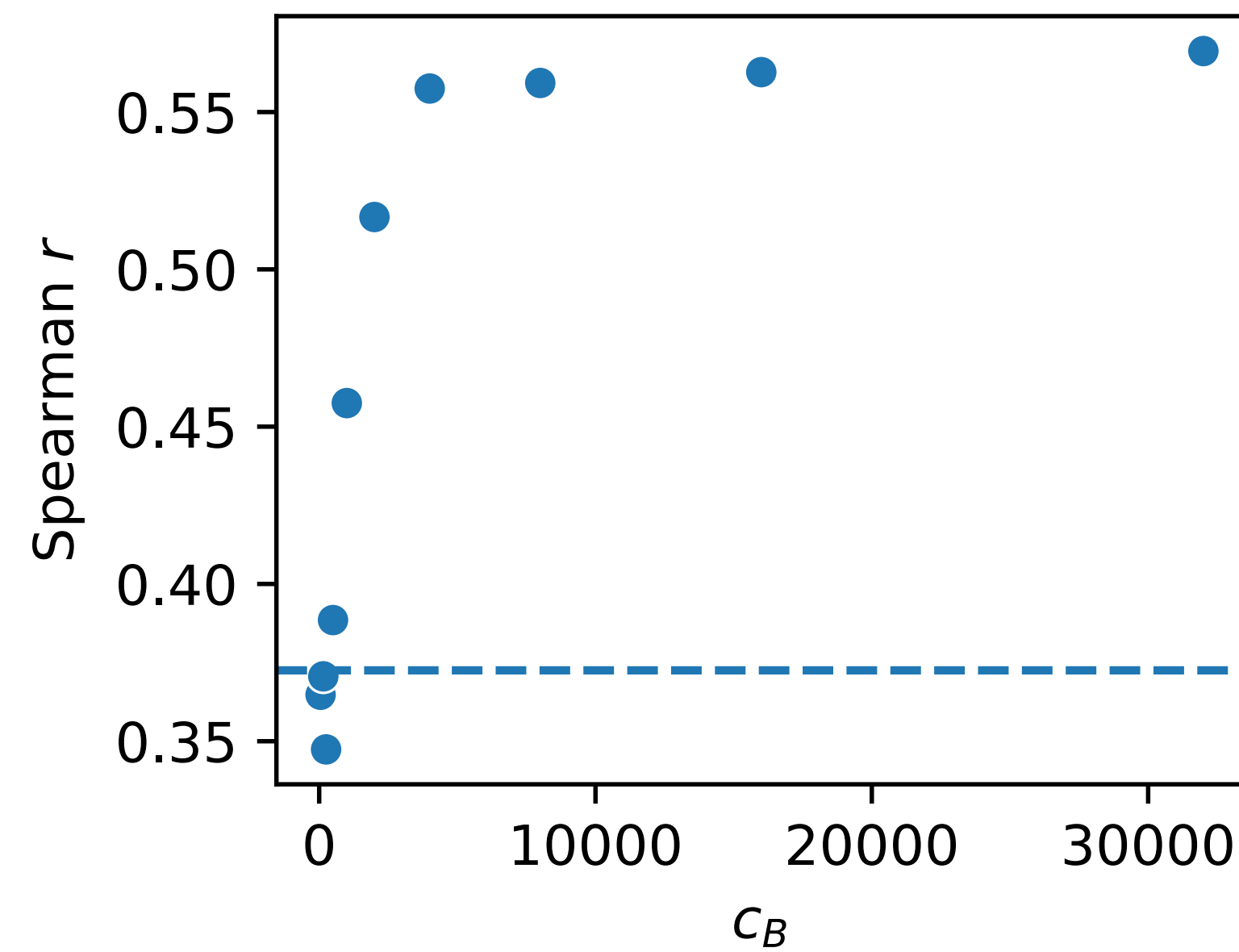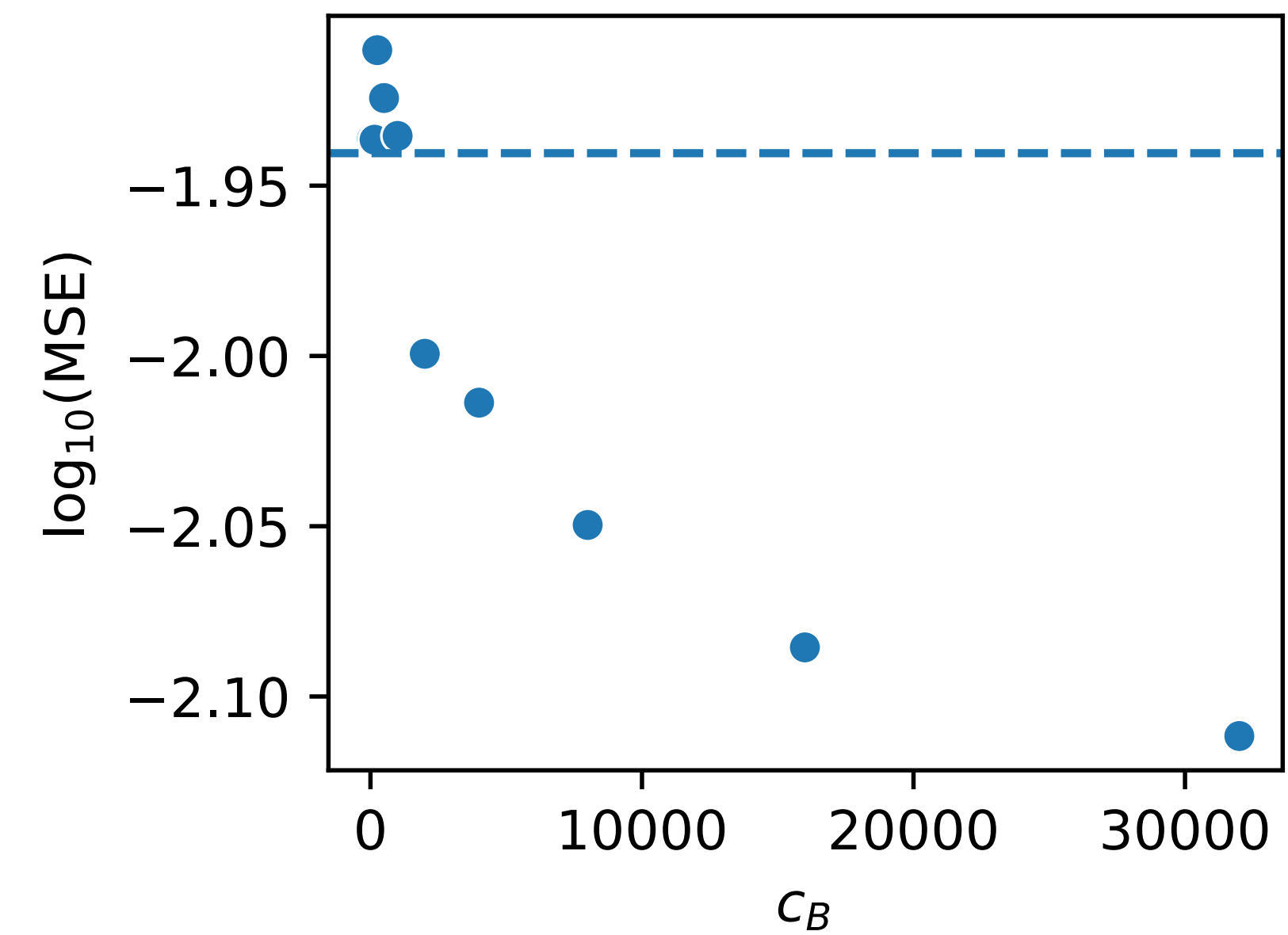

Mouse Skin Genes (ATAC) Topic-Gene

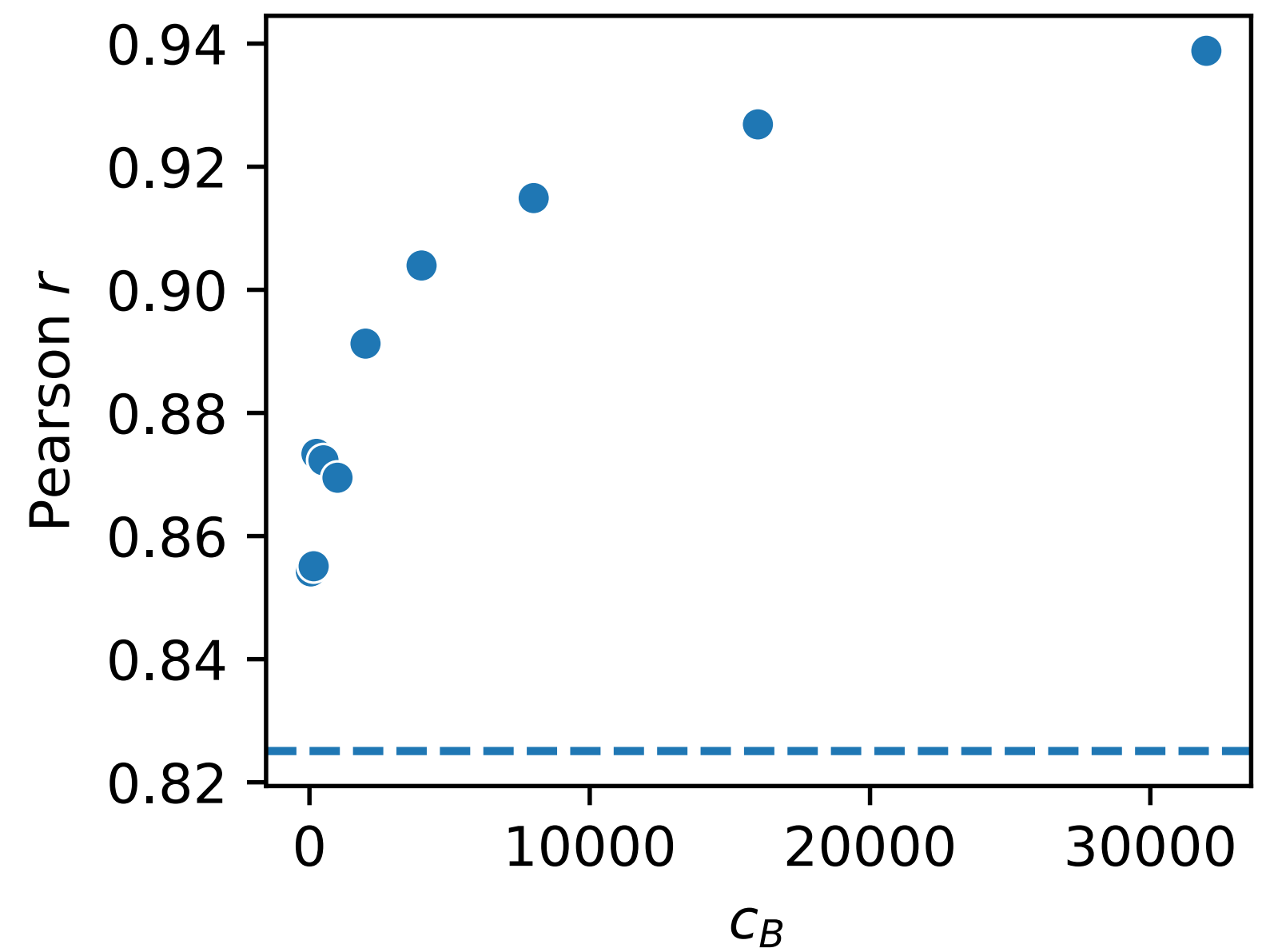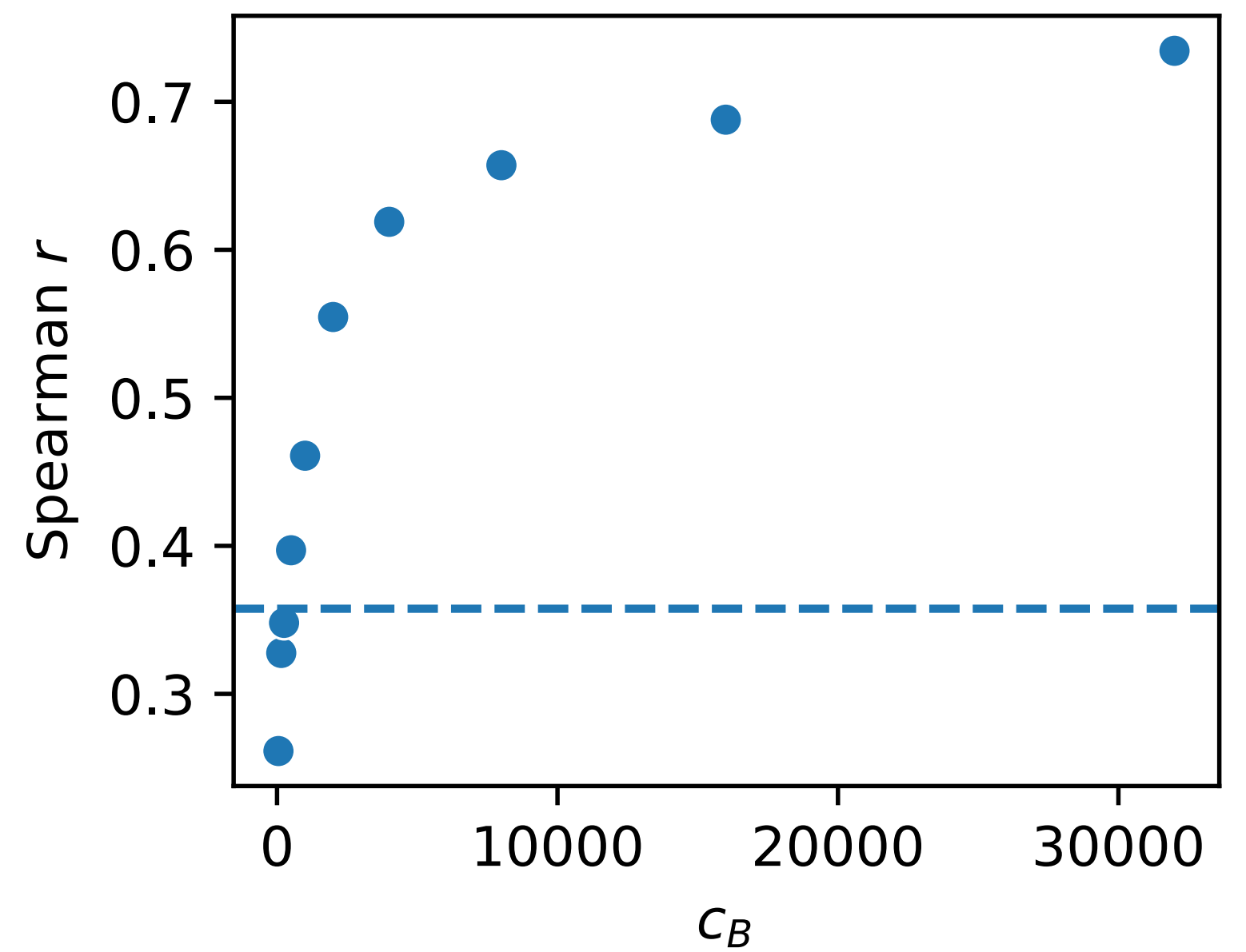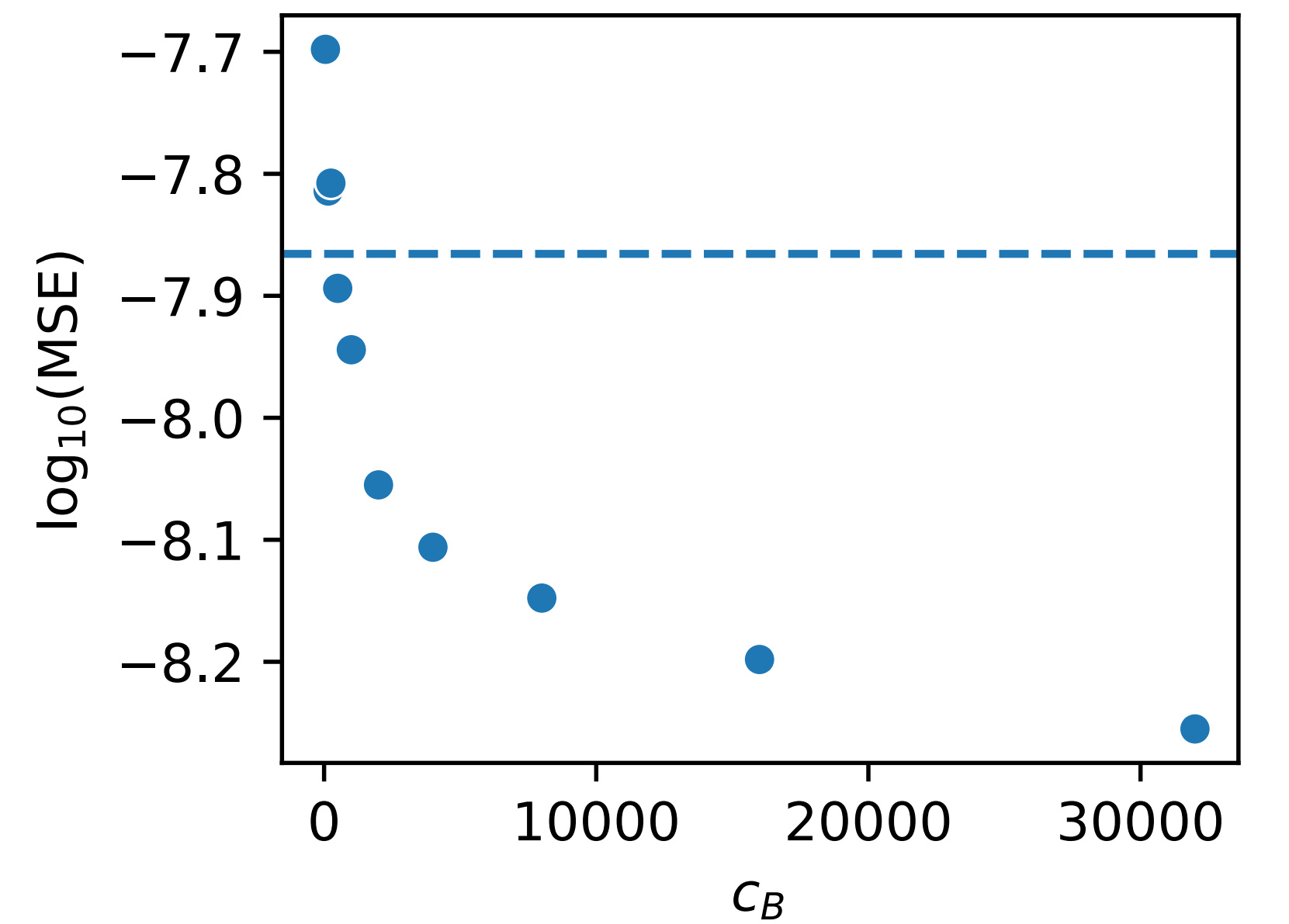

Supplement: S11 Fig — Summary statistics (y-axis) between output matrices of the joint model and LDA with matrix prior, both trained on the SHARE-seq mouse skin scATAC-seq data with cut sites summed over genes (i.e. using the genes vocabulary), show that as the weight of the prior increases, agreement between the matrix prior and joint model also increases. Each plot shows the matrix prior LDA results (points) for increasing values of cB (x-axis) versus the uniform prior (blue dotted line). The top row of plots shows summary statistics for the cell-topic matrix, and the bottom row of plots shows summary statistics for the topic-gene matrix. (PDF) [file pcbi.1011049.s014.pdf]

Mouse Skin Genes (RNA) Cell-Topic

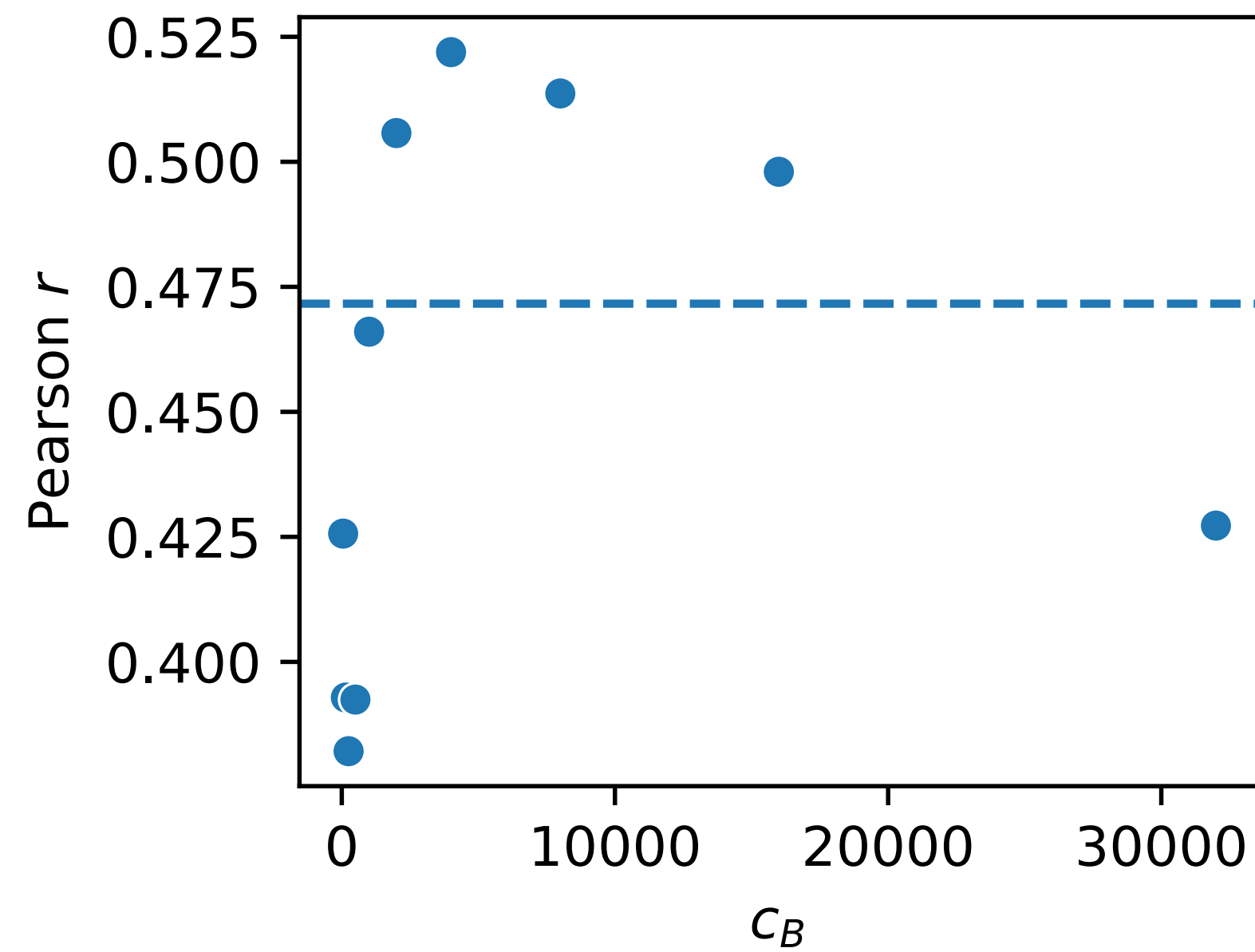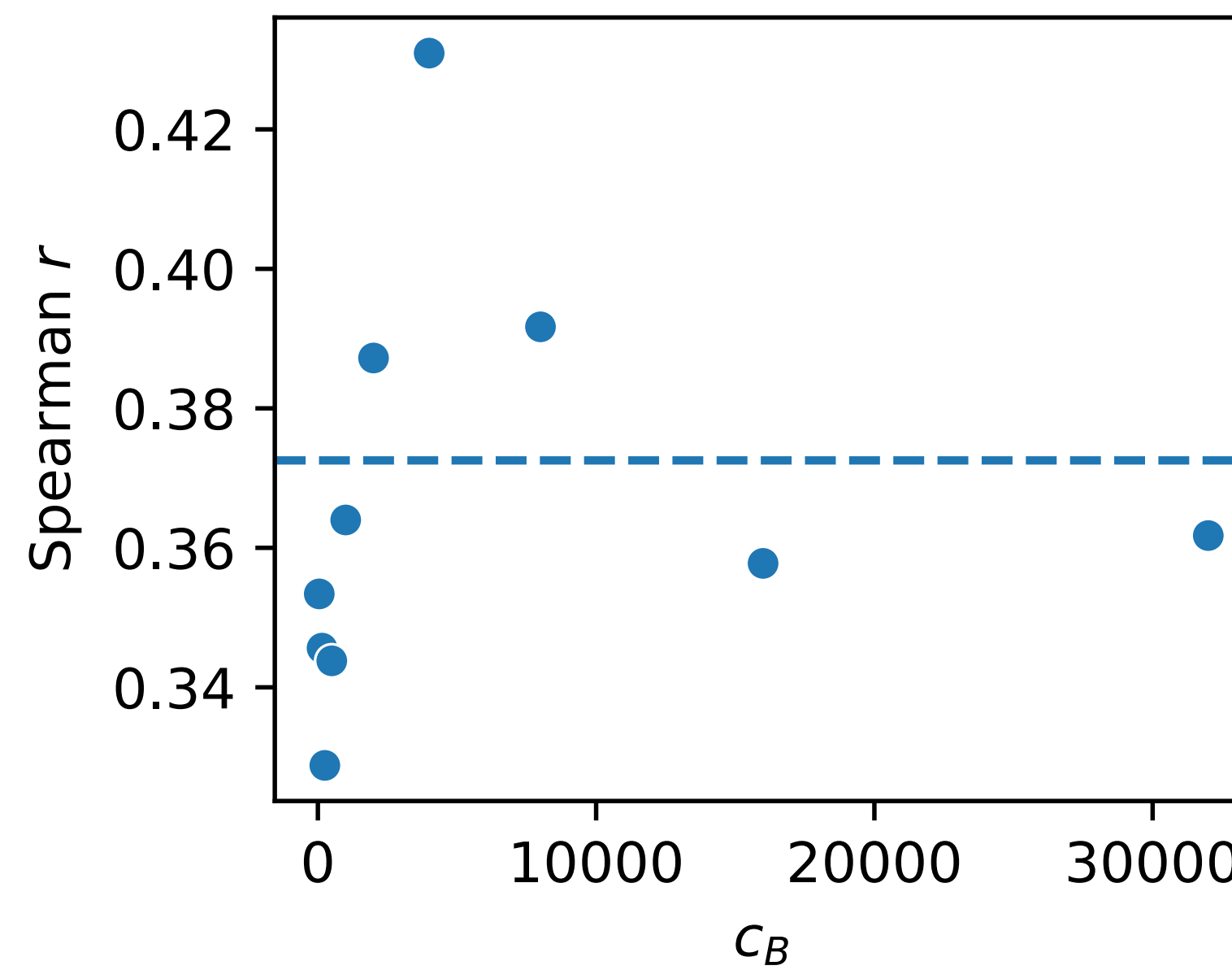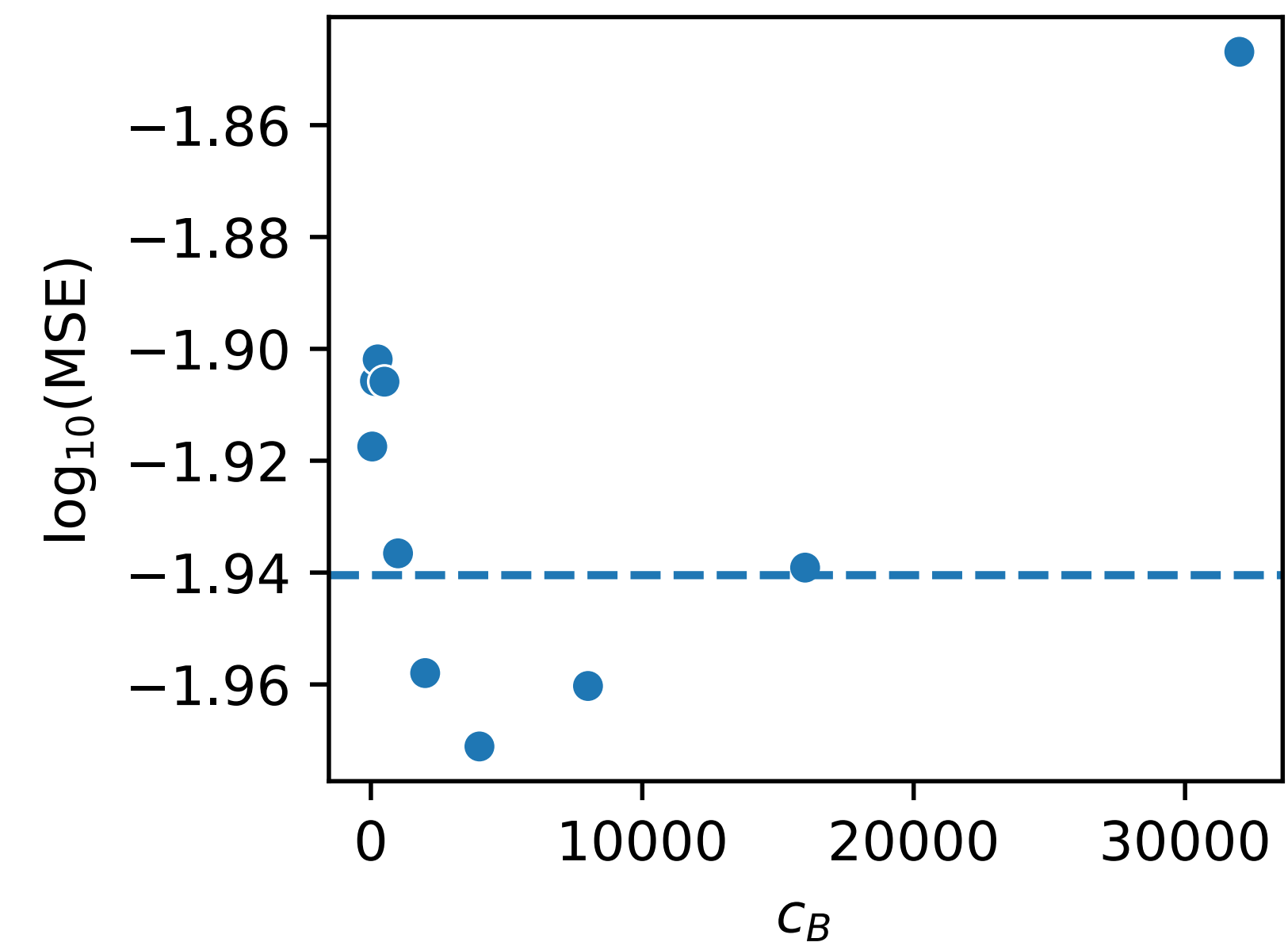

Mouse Skin Genes (RNA) Topic-Gene

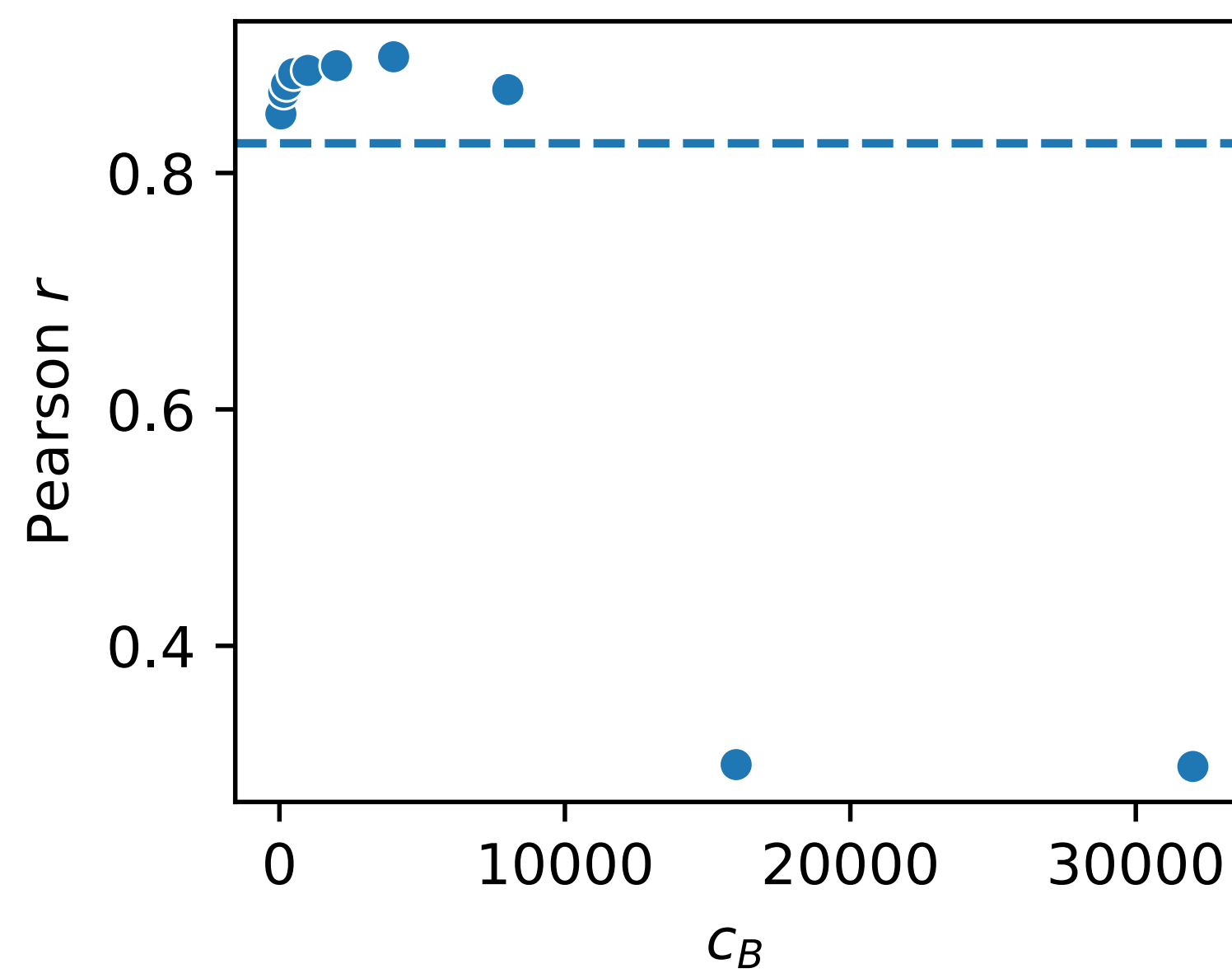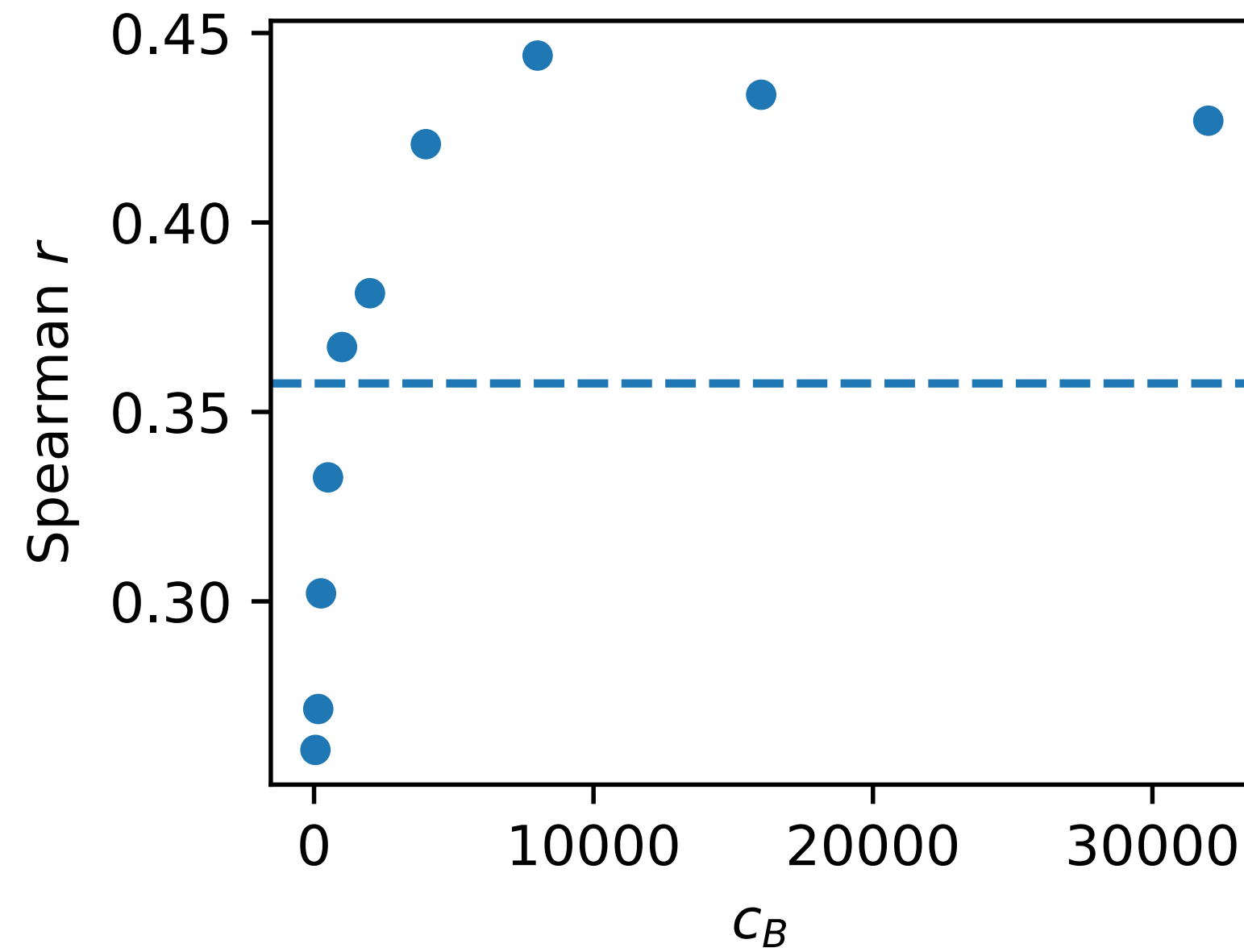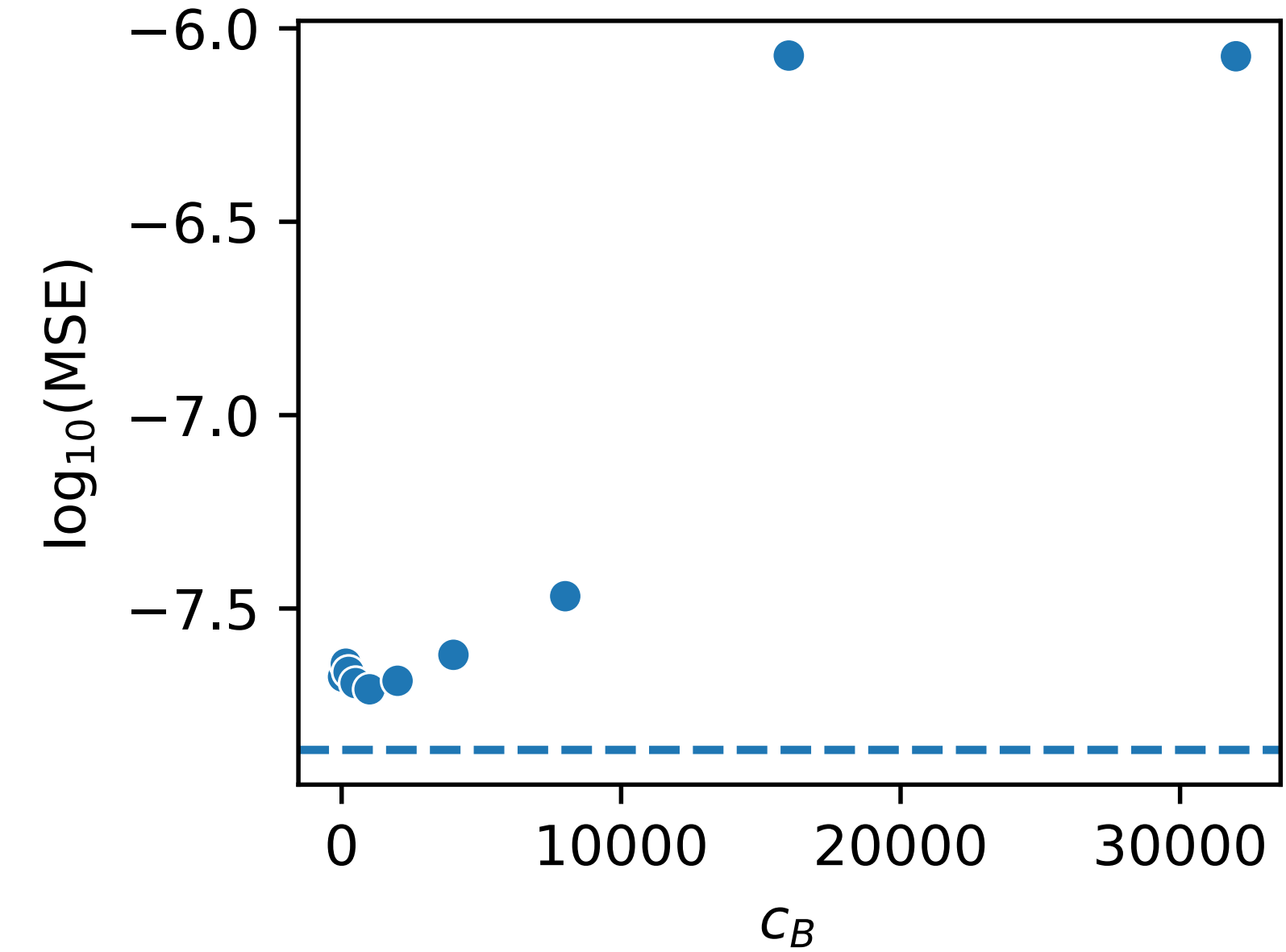

Supplement: S12 Fig — Summary statistics (y-axis) between output matrices of the joint model and LDA with matrix prior, both trained on the SHARE-seq mouse skin scRNA-seq data, show that as the weight of the prior increases, agreement between the matrix prior and joint model also increases with moderate weights and declines with higher weights. Each plot shows the matrix prior LDA results (points) for increasing values of cB (x-axis) versus the uniform prior (blue dotted line). The top row of plots shows summary statistics for the cell-topic matrix, and the bottom row of plots shows summary statistics for the topic-gene matrix. (PDF) [file pcbi.1011049.s015.pdf]

Mouse Skin Peaks Cell-Topic

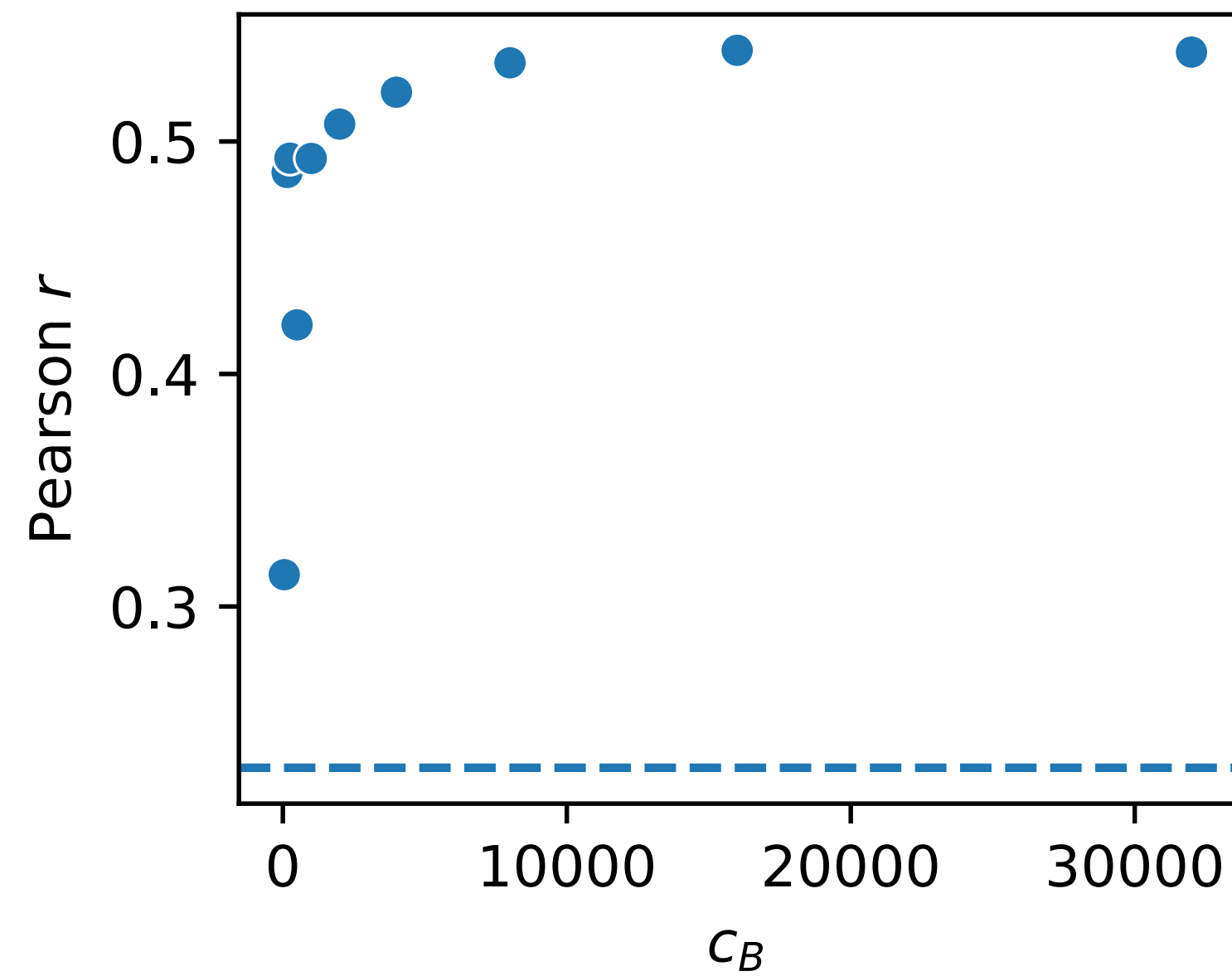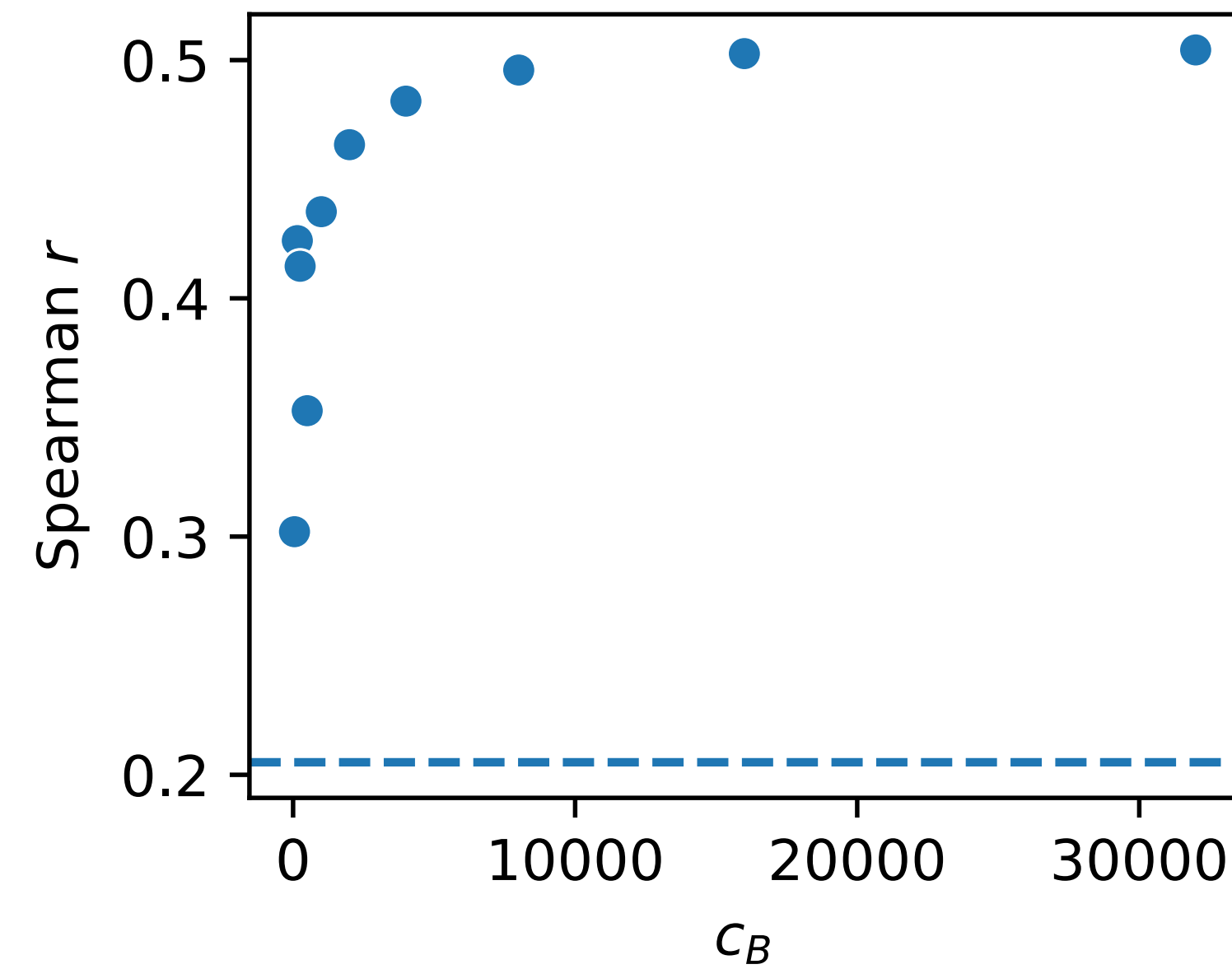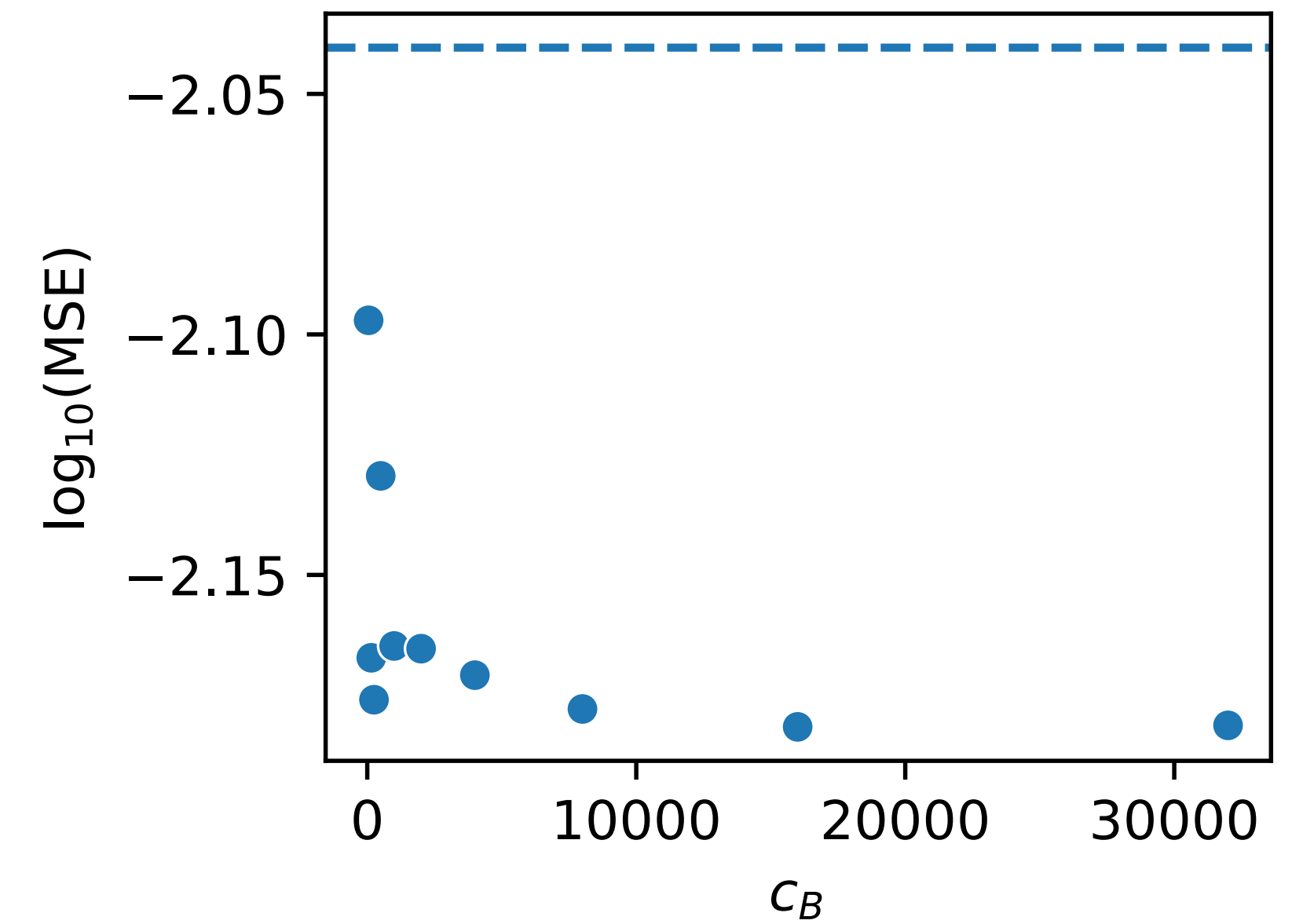

Mouse Skin Peaks Topic-Gene

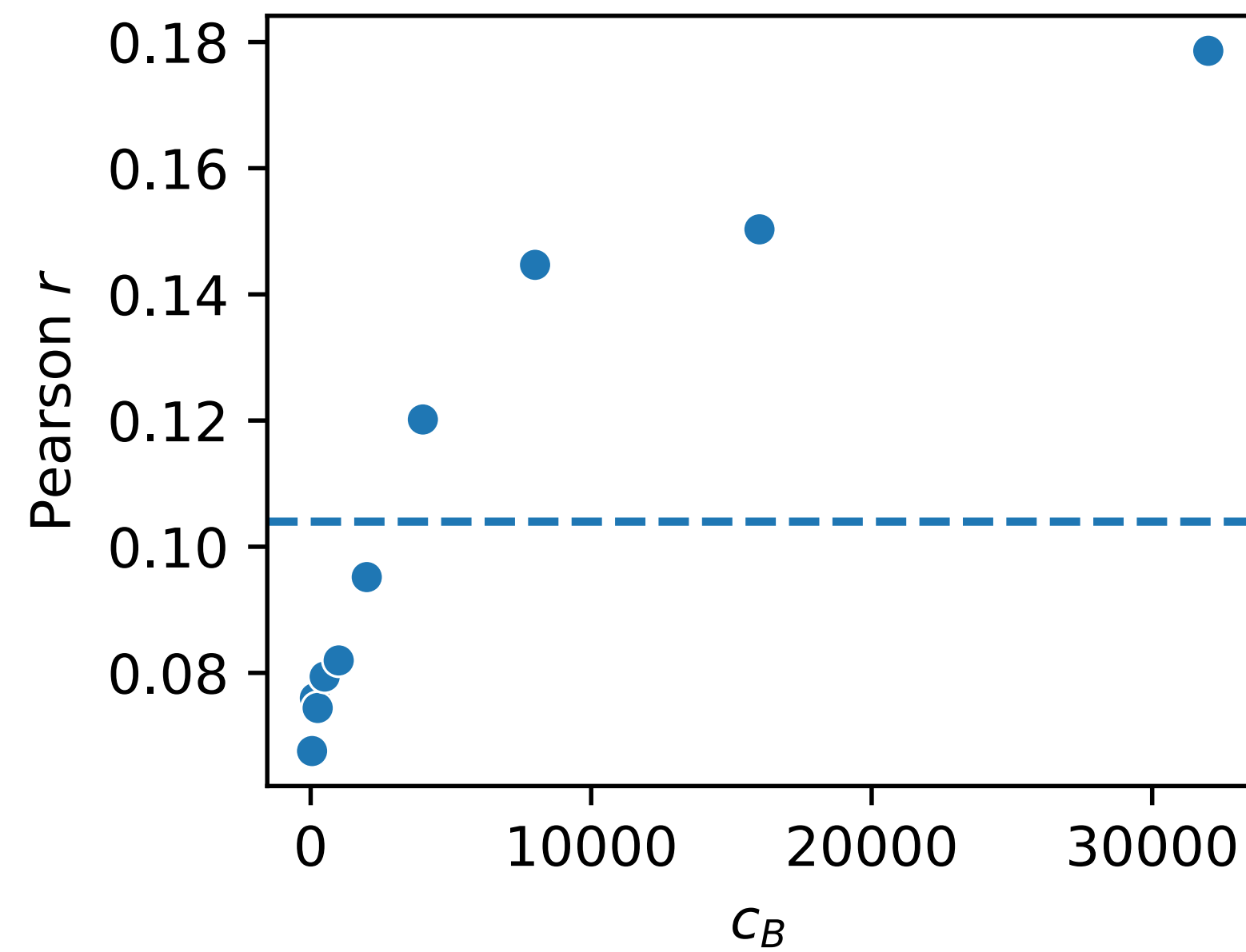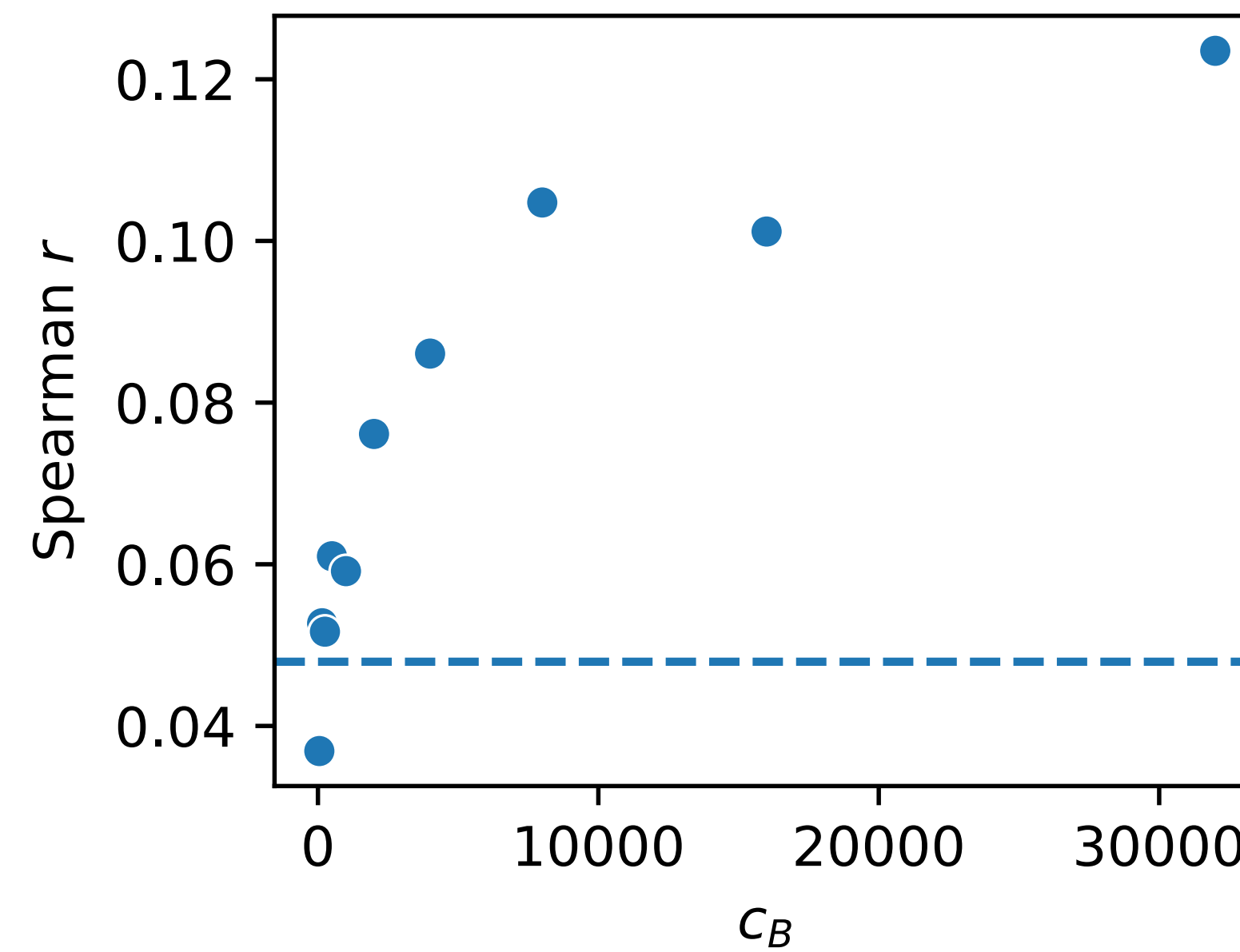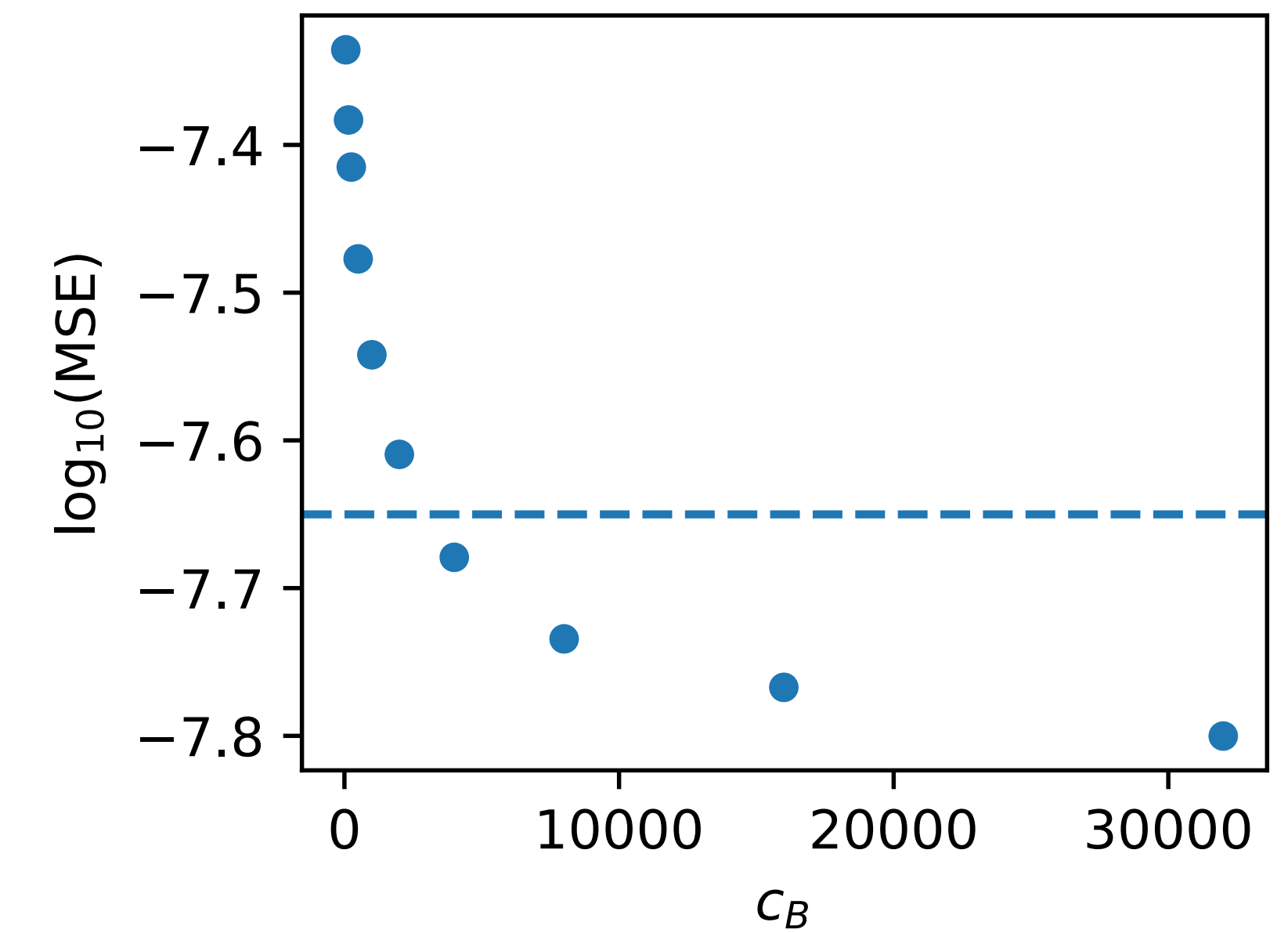

Supplement: S13 Fig — Summary statistics (y-axis) between output matrices of the joint model and LDA with matrix prior, both trained on the SHARE-seq mouse skin scATAC-seq data (i.e. using the peaks vocabulary), show that as the weight of the prior increases, agreement between the matrix prior and joint model also increases. Each plot shows the matrix prior LDA results (points) for increasing values of cB (x-axis) versus the uniform prior (blue dotted line). The top row of plots shows summary statistics for the cell-topic matrix, and the bottom row of plots shows summary statistics for the topic-peak matrix. (PDF) [file pcbi.1011049.s016.pdf]

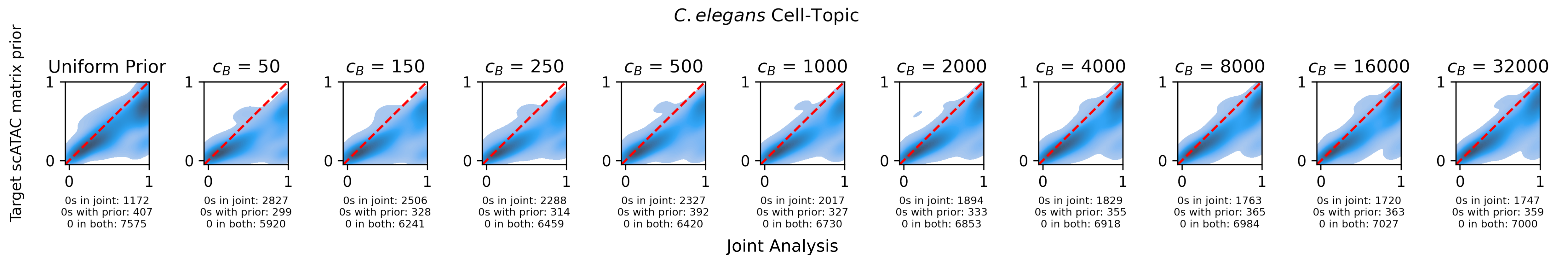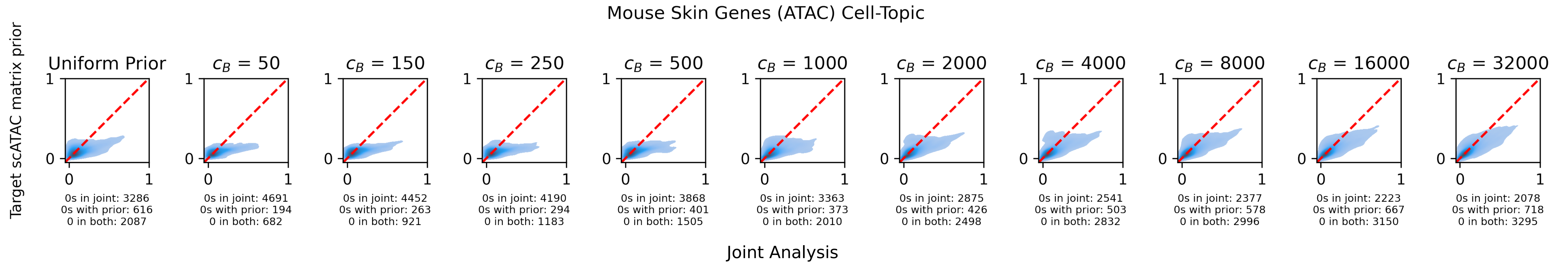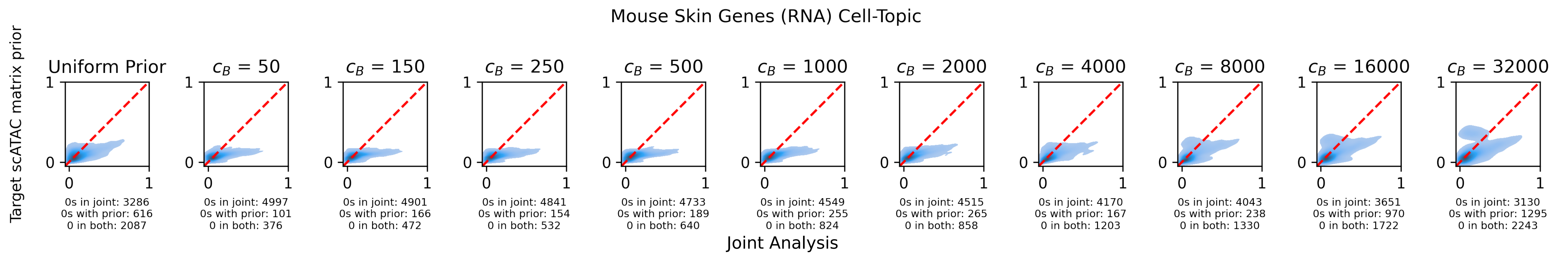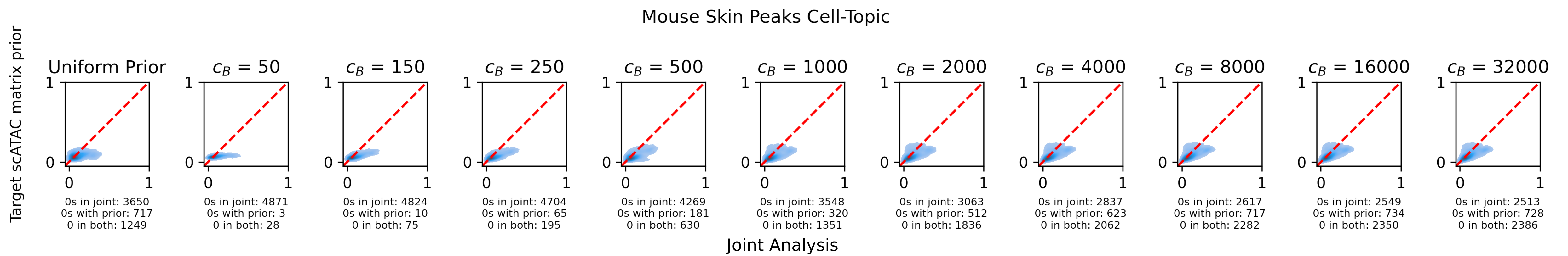

Supplement: S14 Fig — Comparing the cell-topic matrices of the joint model versus the LDA with the matrix prior reveals that as the weight of the matrix prior increases, the agreement between the models increases. The effect of different values of cB were evaluated by comparing the cell-topic matrix using the matrix prior to the cell-topic matrix from the joint model. Different values of cB are plotted across different columns, and different datasets are shown in different rows. We first flatten the cell-topic matrices so that they can be plotted. The cell-topic assignments from the joint model are shown on the x-axis, and the inferred cell-topic assignments from the matrix prior LDA are shown on the y-axis. A dotted red line is drawn to indicate the line y = x. Zero values are omitted from the plots, but the number of zeros exclusively in the cell-topic matrix of the joint model, exclusively in the cell-topic matrix of the LDA with matrix prior, and number of zeros in both is noted below each plot. (PDF) [file pcbi.1011049.s017.pdf]

Silhouette

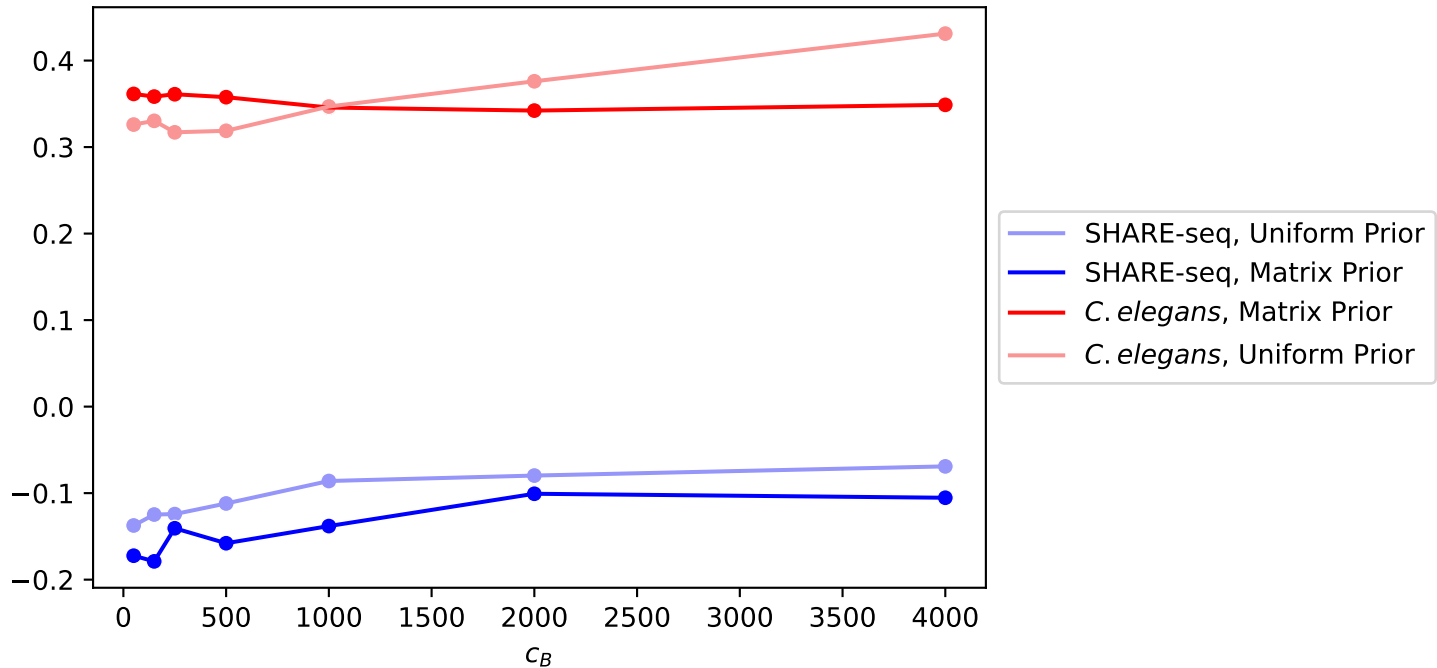

Supplement: S16 Fig — The matrix prior and uniform prior show similar performance as a function of cB. Average silhouette values for the published cell type annotations (y-axis) are plotted against increasing values of cB (x-axis). Different colored lines indicate whether the SHARE-seq data set (red) or the C. elegans data set (blue) was used. Each data set was analyzed using a uniform prior (lighter colors) and the matrix prior (darker colors). (PDF) [file pcbi.1011049.s019.pdf]

(A) UMAP Plots with  $\alpha = 0.30$ , Topics = 15

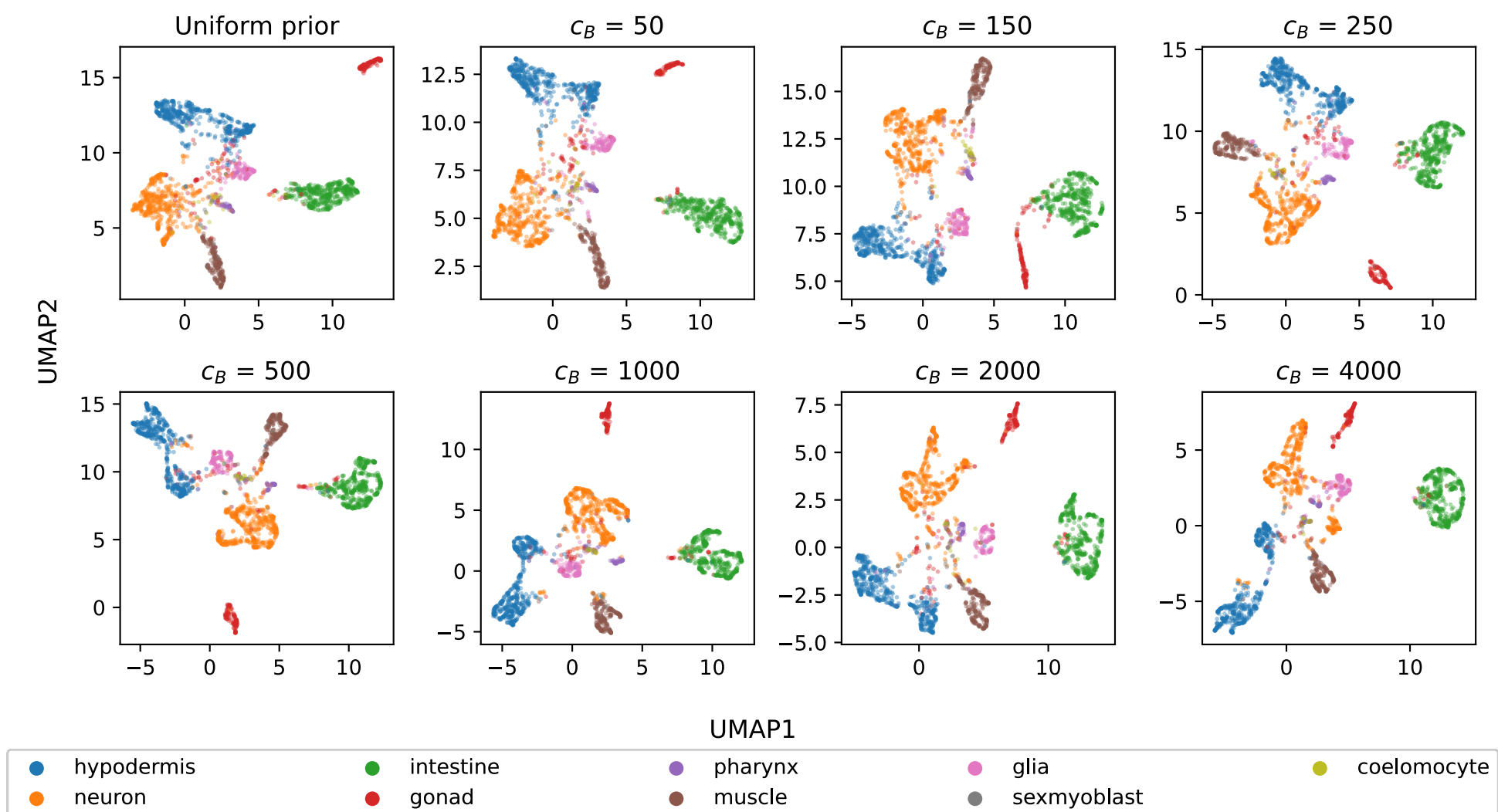

(B) Silhouette Plots with  $\alpha = 0.30$ , Topics = 15

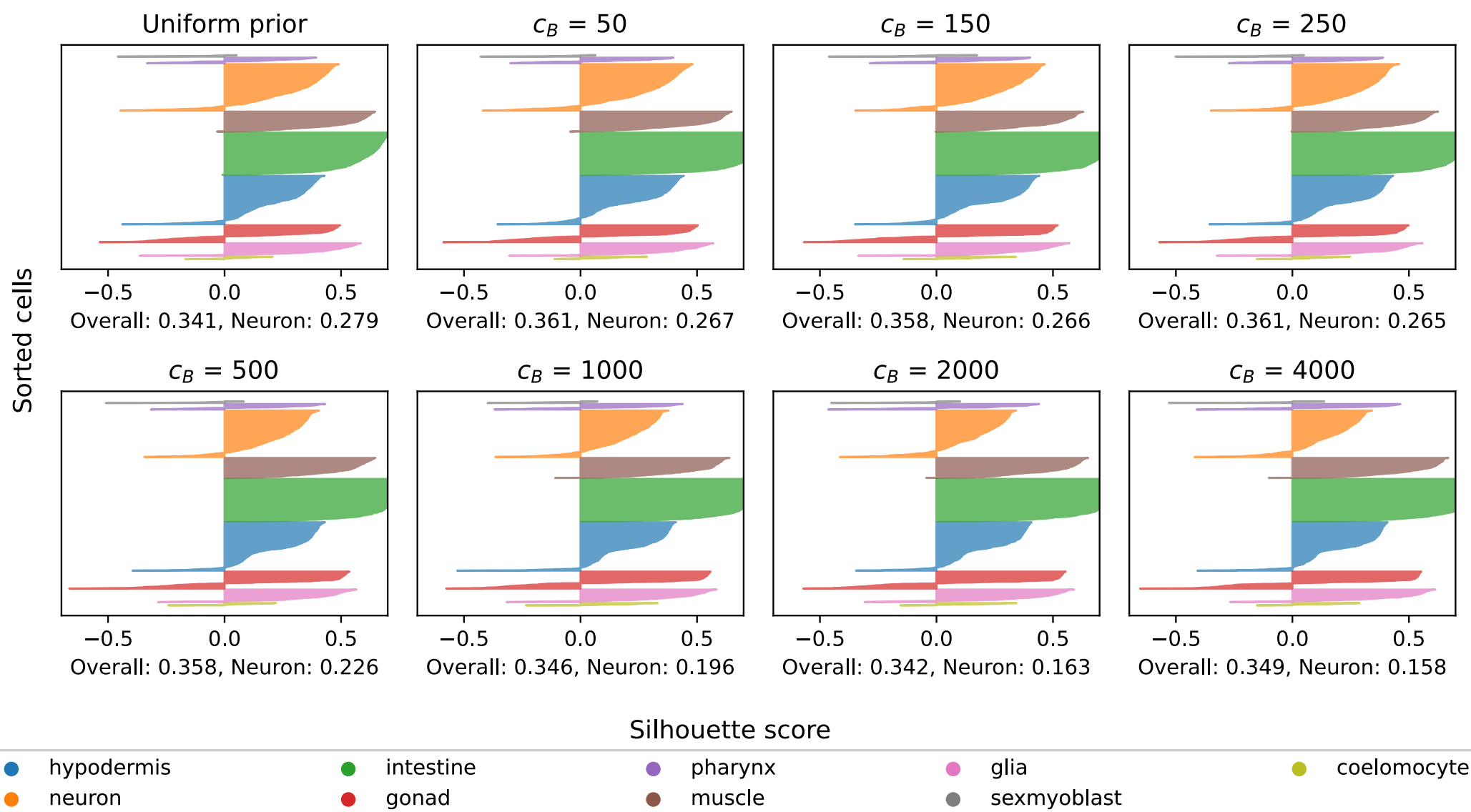

Supplement: S17 Fig — A: UMAP plot of all the C. elegans data reveal cell type structure from LDA analysis with different weights cB of the matrix prior. Cells are colored based on their published cell type annotations. B: Silhouette plots demonstrate that in C. elegans, the silhouette value did not improve with increased weight of the prior. Silhouette values are shown for C. elegans cell types plotted for results from a uniform prior LDA model and matrix prior LDA models trained with increasing values of cB using 15 topics and the scATAC-seq data translated into the genes vocabulary (ATAC cut sites summed over the promoter and gene body for 13,734 genes). Each row in each plot represents one cell, and the silhouette value of the cell is the length of the line. The mean silhouette value for all of the cells is shown as “Overall”, and the mean silhouette value for only the neurons is shown as “Neuron.” (PDF) [file pcbi.1011049.s020.pdf]

(A) UMAP Plots with  $\alpha = 0.30$ , Topics = 15

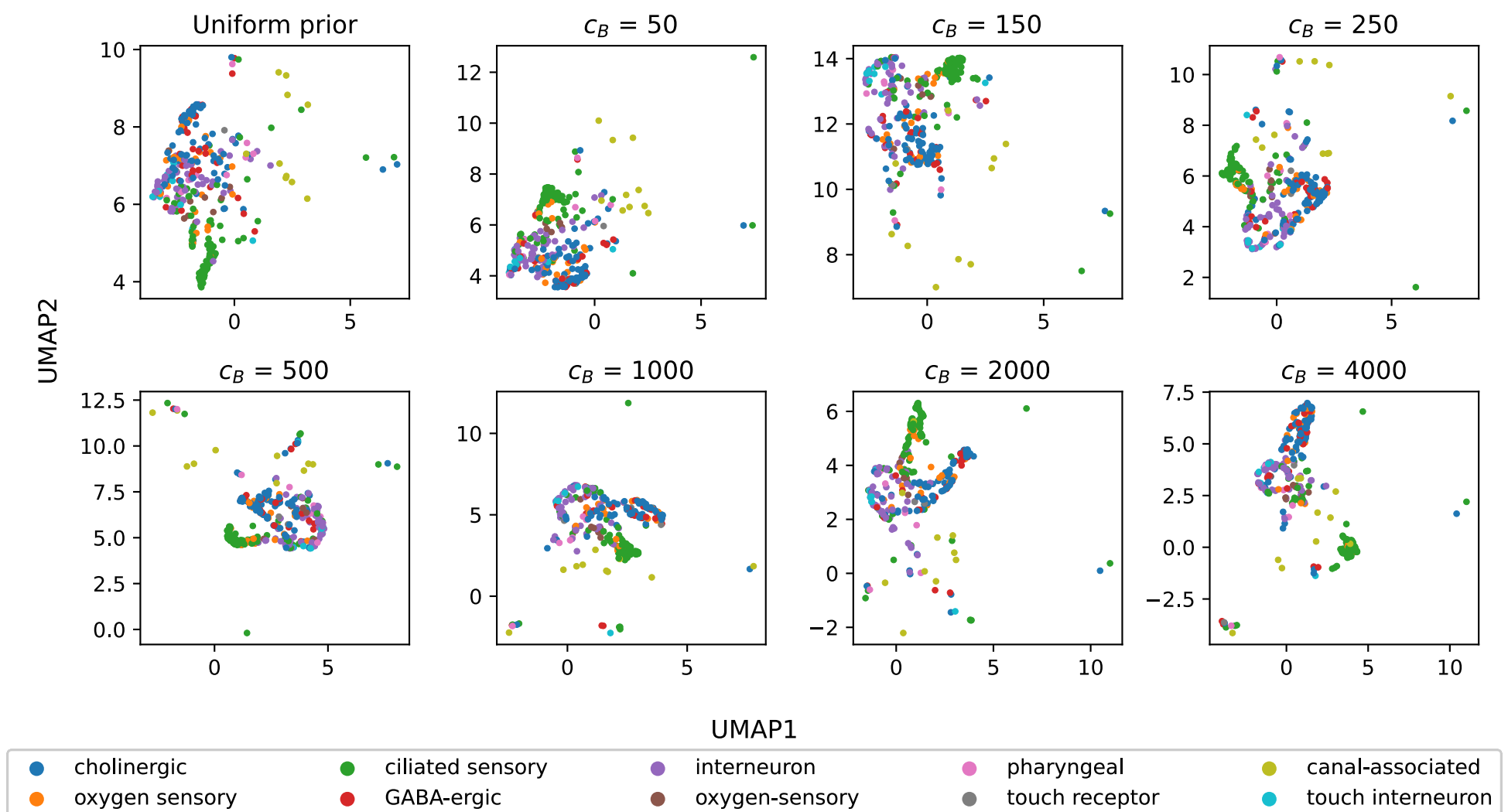

(B) Silhouette Plots with  $\alpha = 0.30$ , Topics = 15

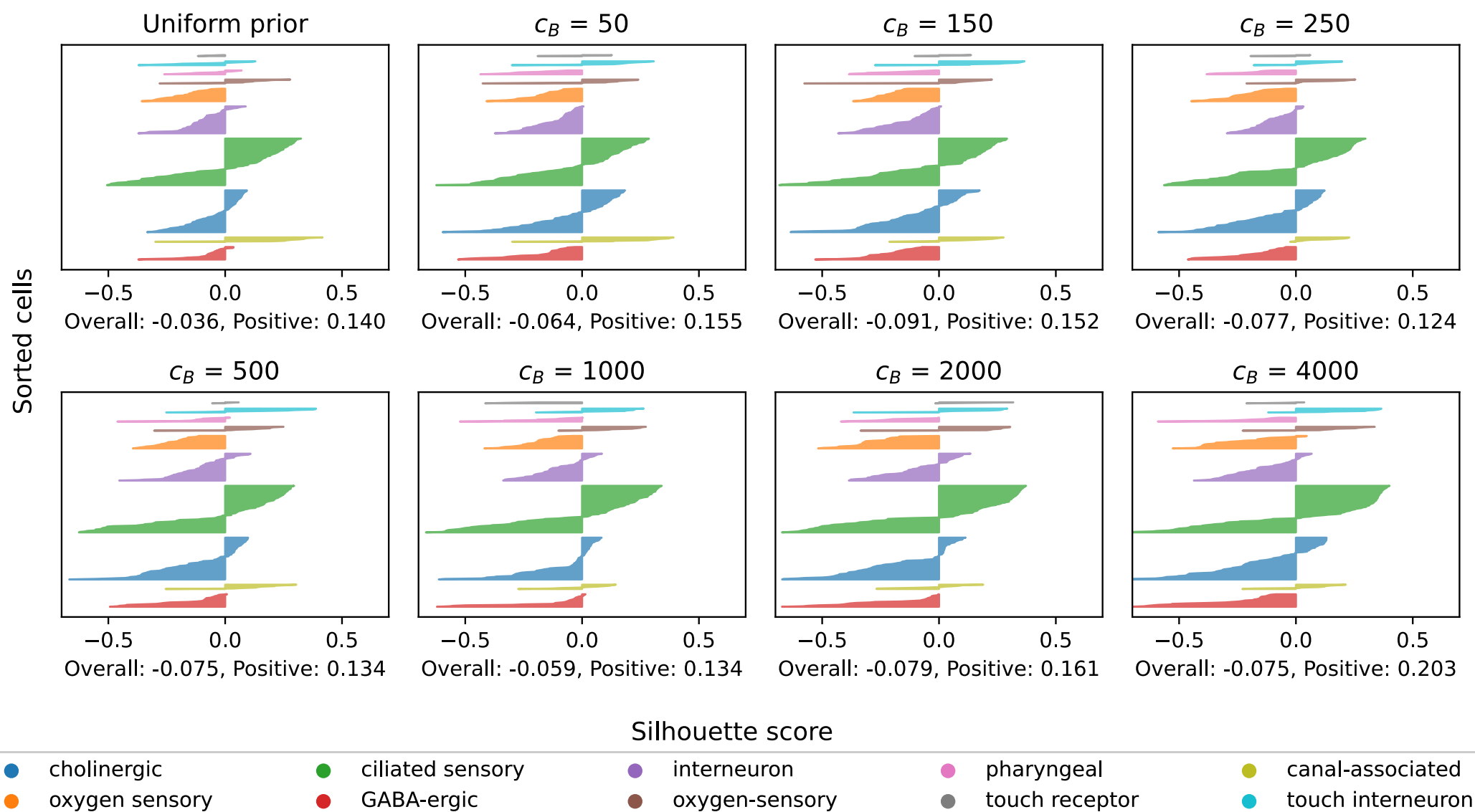

Supplement: S18 Fig — A: A subset of S17 Fig (a) that includes only neurons, demonstrating that increased values of cB have little effect on the ability of the matrix prior LDA to distinguish among published cell types. Cells are colored by published neuron subtype labels. B: Silhouette plots of the neurons in the C. elegans dataset. The overall mean silhouette values and the mean positive silhouette values are reported. (PDF) [file pcbi.1011049.s021.pdf]
