# Supplementary material for: Matrix prior for data transfer between single cell data types in latent Dirichlet allocation
Source: PLoS Comput Biol. 2023 May 5;19(5):e1011049. doi: 10.1371/journal.pcbi.1011049 (PMC10191269; doi:10.1371/journal.pcbi.1011049)
Supplement: S15 Fig — Comparing the topic-gene matrices of the joint model versus the LDA with the matrix prior reveals that as the weight of the prior increases, the agreement between the models increases. The effect of different values of cB were evaluated by comparing the topic-gene matrix using the matrix prior to the topic-gene matrix from the joint model. Different values of cB are plotted across different columns, and different datasets are shown in different rows. We first flatten the topic-gene matrices so that they can be plotted. The topic-gene assignments from the joint model are shown on the x-axis, and the inferred topic-gene assignments from the matrix prior LDA are shown on the y-axis. A dotted red line is drawn to indicate the line y = x. Zero values are omitted from the plots, but the number of zeros exclusively in the topic-gene matrix of the joint model, exclusively in the topic-gene matrix of the LDA with matrix prior, and number of zeros in both is noted below each plot. (PDF) [file pcbi.1011049.s018.pdf]

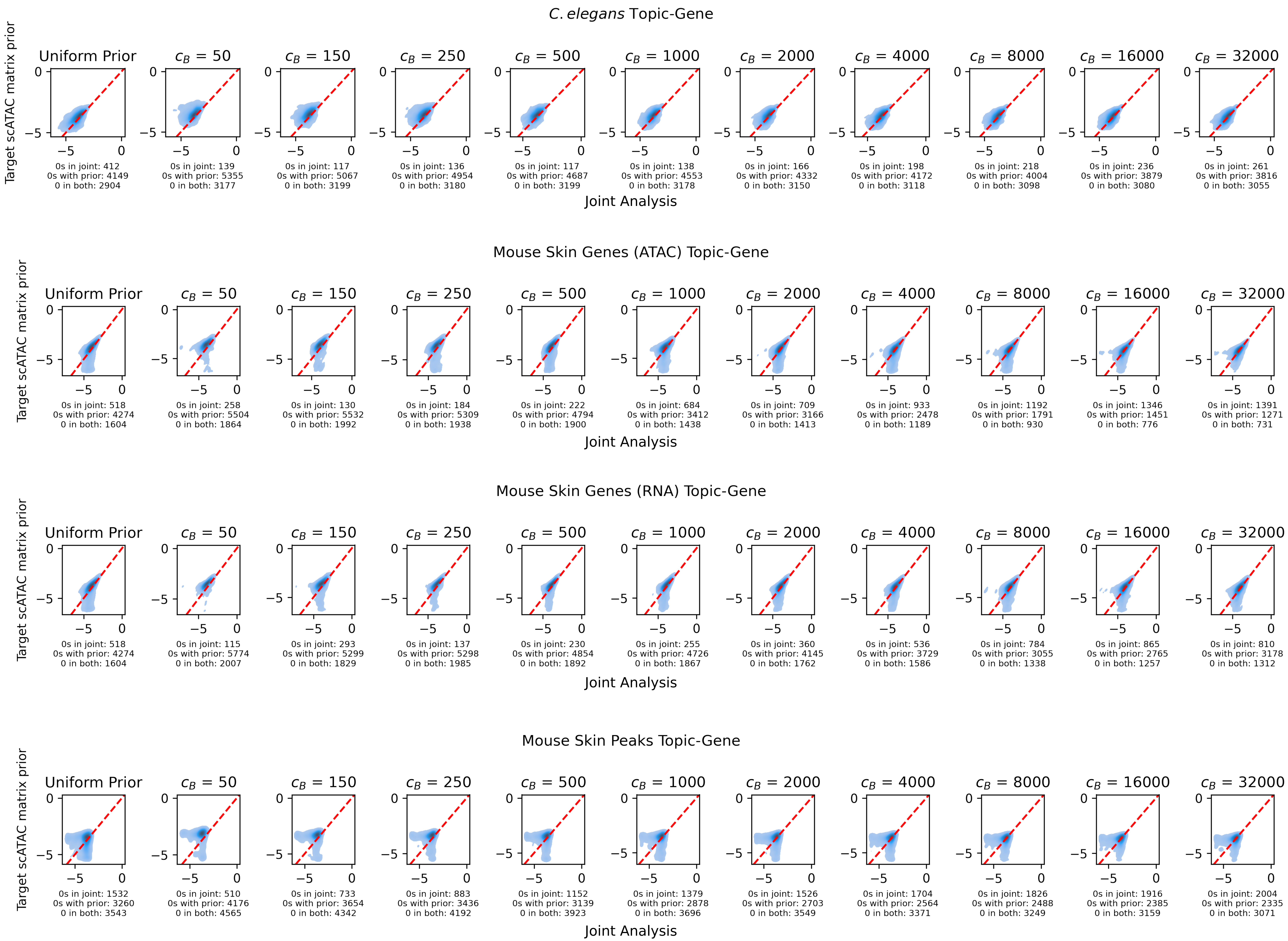

C. elegans Topic-Gene
Target scATAC matrix prior
Uniform Prior
c_B = 50
c_B = 150
c_B = 250
c_B = 500
c_B = 1000
c_B = 2000
c_B = 4000
c_B = 8000
c_B = 16000
c_B = 32000
0s in joint: 412 0s with prior: 4149 0 in both: 2904
0s in joint: 139 0s with prior: 5355 0 in both: 3177
0s in joint: 117 0s with prior: 5067 0 in both: 3199
0s in joint: 136 0s with prior: 4954 0 in both: 3180
0s in joint: 117 0s with prior: 4687 0 in both: 3199
0s in joint: 138 0s with prior: 4553 0 in both: 3178
0s in joint: 166 0s with prior: 4332 0 in both: 3150
0s in joint: 198 0s with prior: 4172 0 in both: 3118
0s in joint: 218 0s with prior: 4004 0 in both: 3098
0s in joint: 236 0s with prior: 3879 0 in both: 3080
0s in joint: 261 0s with prior: 3816 0 in both: 3055
Joint Analysis
Mouse Skin Genes (ATAC) Topic-Gene
Target scATAC matrix prior
Uniform Prior
c_B = 50
c_B = 150
c_B = 250
c_B = 500
c_B = 1000
c_B = 2000
c_B = 4000
c_B = 8000
c_B = 16000
c_B = 32000
0s in joint: 518 0s with prior: 4274 0 in both: 1604
0s in joint: 258 0s with prior: 5504 0 in both: 1864
0s in joint: 130 0s with prior: 5532 0 in both: 1992
0s in joint: 184 0s with prior: 5309 0 in both: 1938
0s in joint: 222 0s with prior: 4794 0 in both: 1900
0s in joint: 684 0s with prior: 3412 0 in both: 1438
0s in joint: 709 0s with prior: 3166 0 in both: 1413
0s in joint: 933 0s with prior: 2478 0 in both: 1189
0s in joint: 1192 0s with prior: 1791 0 in both: 930
0s in joint: 1346 0s with prior: 1451 0 in both: 776
0s in joint: 1391 0s with prior: 1271 0 in both: 731
Joint Analysis
Mouse Skin Genes (RNA) Topic-Gene
Target scATAC matrix prior
Uniform Prior
c_B = 50
c_B = 150
c_B = 250
c_B = 500
c_B = 1000
c_B = 2000
c_B = 4000
c_B = 8000
c_B = 16000
c_B = 32000
0s in joint: 518 0s with prior: 4274 0 in both: 1604
0s in joint: 115 0s with prior: 5774 0 in both: 2007
0s in joint: 293 0s with prior: 5299 0 in both: 1829
0s in joint: 137 0s with prior: 5298 0 in both: 1985
0s in joint: 230 0s with prior: 4854 0 in both: 1892
0s in joint: 255 0s with prior: 4726 0 in both: 1867
0s in joint: 360 0s with prior: 4145 0 in both: 1762
0s in joint: 536 0s with prior: 3729 0 in both: 1586
0s in joint: 784 0s with prior: 3055 0 in both: 1338
0s in joint: 865 0s with prior: 2765 0 in both: 1257
0s in joint: 810 0s with prior: 3178 0 in both: 1312
Joint Analysis
Mouse Skin Peaks Topic-Gene
Target scATAC matrix prior
Uniform Prior
c_B = 50
c_B = 150
c_B = 250
c_B = 500
c_B = 1000
c_B = 2000
c_B = 4000
c_B = 8000
c_B = 16000
c_B = 32000
0s in joint: 1532 0s with prior: 3260 0 in both: 3543
0s in joint: 510 0s with prior: 4176 0 in both: 4565
0s in joint: 733 0s with prior: 3654 0 in both: 4342
0s in joint: 883 0s with prior: 3436 0 in both: 4192
0s in joint: 1152 0s with prior: 3139 0 in both: 3923
0s in joint: 1379 0s with prior: 2878 0 in both: 3696
0s in joint: 1526 0s with prior: 2703 0 in both: 3549
0s in joint: 1704 0s with prior: 2564 0 in both: 3371
0s in joint: 1826 0s with prior: 2488 0 in both: 3249
0s in joint: 1916 0s with prior: 2385 0 in both: 3159
0s in joint: 2004 0s with prior: 2335 0 in both: 3071
Joint Analysis
