# Supplementary material for: Matrix prior for data transfer between single cell data types in latent Dirichlet allocation
Source: PLoS Comput Biol. 2023 May 5;19(5):e1011049. doi: 10.1371/journal.pcbi.1011049 (PMC10191269; doi:10.1371/journal.pcbi.1011049)
Supplement: Attachment — Submitted filename: review_response.pdf [file pcbi.1011049.s022.pdf]

Review response for: Matrix prior for data transfer between single cell data types in latent Dirichlet allocation

Alan Min[1], Timothy Durham[2,3], Louis Gevirtzman[2], and William Stafford Noble[2]

[1]Department of Statistics, University of Washington
[2]Department of Genomics, University of Washington
[3]Broad Institute
[4]Paul G. Allen School of Computer Science and Engineering, University of Washington

March 15, 2023

Summary

We thank the reviewers for their helpful comments. Below, we have responded to the reviewer comments and where applicable, we have included the original text in green and the revised text in blue.

Reviewer 1

The authors developed a latent Dirchlet allocation-based method, which could leverage information from previously generated large scale single cell genomics data to guide the analysis of new single cell data. The approach is useful for the new data annotation. The authors illustrated the method's ability on both simulation and real datasets. Below are the specific comments:

1. Among all the cases analyzed, the cell types in the reference sets and target sets are the same. However, there might be some target set-specific cell types in the newly generated single cell data, especially, the data generated with different conditions such as control and treatment experiments. How is the method's performance on such data? Could this method capture target set-specific cell types with new topic? This is very important when applying to new data.

We agree that this is an important use case for our method, and we approached this question using the SHARE-seq data in the following way. We used the same target/reference split as in the main text, with 7000 cells in the reference dataset and 700 cells in the target dataset. We then tabulated the number of cells of each cell type, and we systematically removed the top 1, 2, 3, or 4 of the most common cell types from the reference data (Table R1). We additionally tried removing cell types that appeared to be different from other cells (cell types marked as black in Figure R1). In each case, the target data was kept intact. We used

Celltype	Count
Basal	1388
Infundibulum	737
TAC-1	644
Spinous	544
Mix	455

Table R1: Table of the top 5 common cell types in the reference data

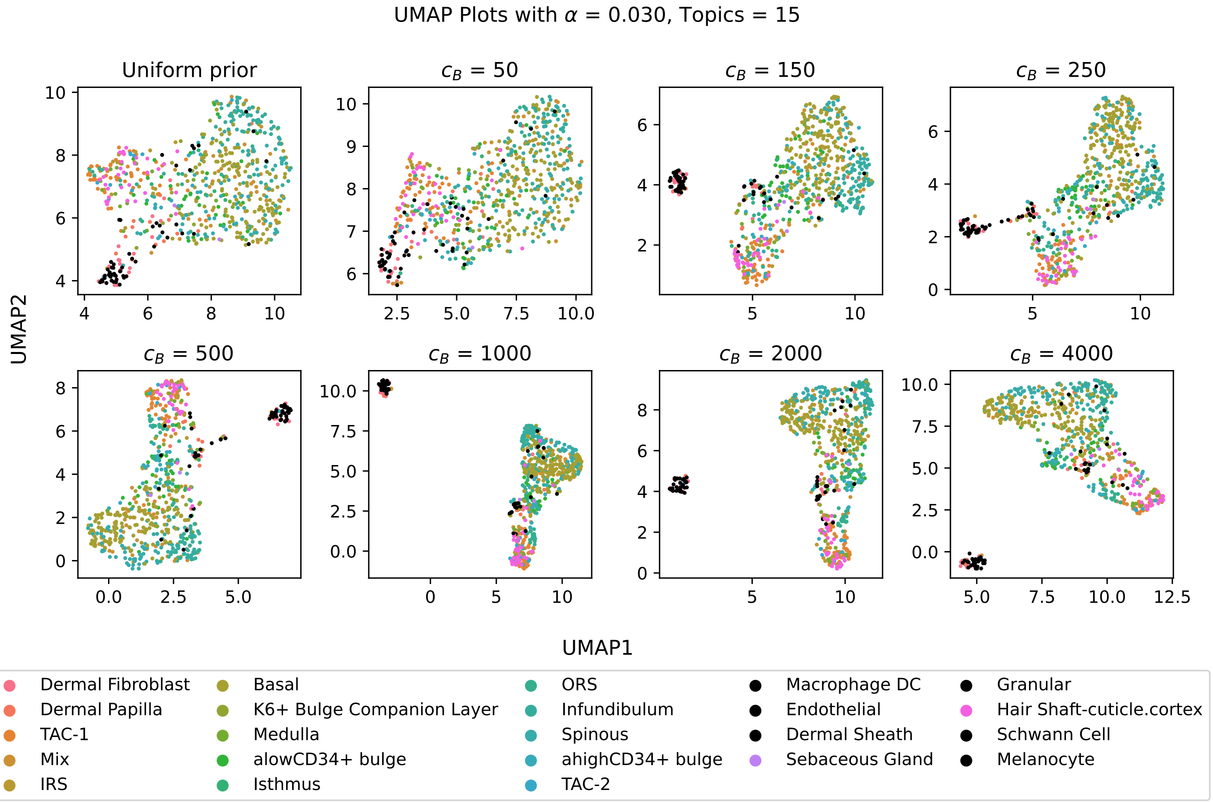

Figure R1: UMAP plots corresponding to Figure 4 from the main text. Cell types are considered ”special” cell types if they are in colored black.

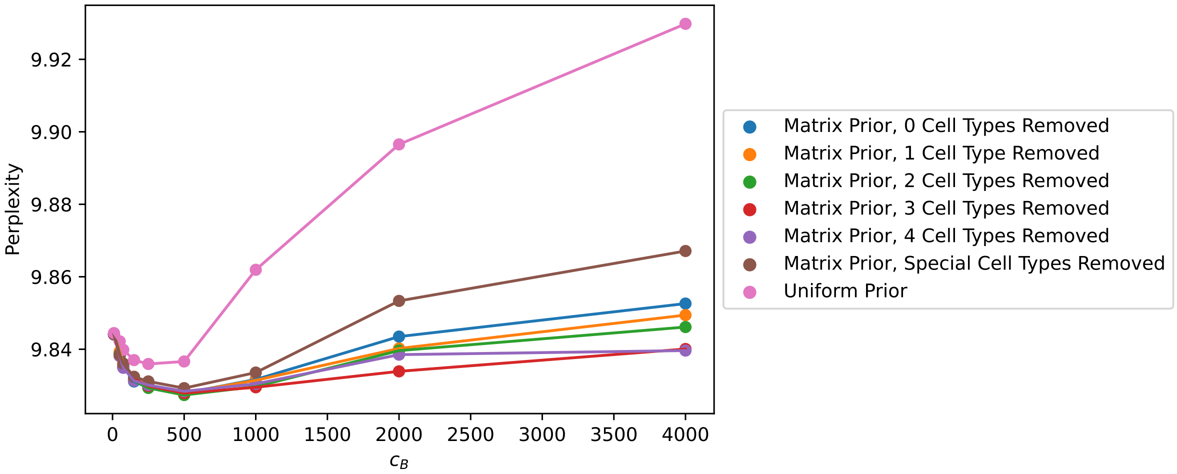

Figure R2: Cell types were removed from the reference dataset in order by population (colors). Perplexity analysis was conducted for each dataset, and the mean perplexity value across different folds (Y-axis) is shown for different values of the concentration parameter c_B (X-axis).

each of these modified reference datasets to create four different priors for the target data, then varied the concentration prior of the prior, c_B, in an LDA of the target data.

Using these priors, we looked at the performance of our method with these cell types removed using

the Chib style perplexity evaluation method (Wallach et al., 2009). In brief, we start by applying the LDA analysis to each of the four reference datasets with different numbers of cell types removed. Then we split the target data set into 90% training and 10% test, and we used the Chib style method to evaluate on the test set. We repeated this procedure for 10 different splits of the target data set across different values of the concentration parameter c_B. This procedure results in one perplexity value for each fold, c_B, and number of removed cell types. We report the average of the perplexity values across each of the 10 folds (Figure R2). Overall, we observe that the perplexity was comparable between the different numbers of cell types that were removed, and better than when the uniform prior was used. Removing the common cell types resulted in perplexity values similar to when no cell types were removed. On the other hand, when the special cell types were removed, the perplexity increased compared to removing common cell types.

2. In section 3.3.2, the procedure to make the comparison between the matrix prior and the joint model was not clearly described. Could you please make it clearer?

We have added some new text to try and make it clear what we are comparing. The original text and revised versions are shown below.

Unlike our simulation experiments, in which we know the true underlying topic distributions, we do not have a ground truth to compare against for our analyses of datasets derived from biological experiments. Instead, to evaluate the performance of our LDA models, we compared the output of each LDA on a target subset to the LDA output when training on the full data set (i.e. the "joint model"), with the interpretation that if our matrix prior allows the smaller target data set LDA to infer similar topics to the joint model, then our matrix prior has succeeded. To make this comparison, we selected just the rows in the cell-topic matrix of the joint model that corresponded to the target dataset cells, we applied our greedy topic matching algorithm to account for any topic-switching that occurred in the LDA of the reference data set compared to the joint model, and then we evaluated the similarity between the outputs of the matrix prior LDA and the joint model using Pearson's correlation, Spearman's correlation, and MSE. We focus on Pearson correlation in the main text but report other metrics in the supplementary Material.

Unlike our simulation experiments, in which we know the true underlying topic distributions, we do not have a ground truth to compare against for our analyses of datasets derived from biological experiments. Instead, to evaluate the performance of our LDA models, we compared the output of each LDA on a target subset to the LDA output when training on the full data set (i.e. the "joint model"). This has the interpretation that if our matrix prior allows the smaller target data set LDA to infer similar topics to the joint model, then our matrix prior has succeeded. **The joint model results in topic assignments for all cells, inclusive of both cells assigned to be reference cells and those assigned to be target cells. Hence, to compare the joint model with the target LDA**, we selected the rows in the cell-topic matrix of the joint model that corresponded to the target dataset cells. We then applied our greedy topic matching algorithm to account for any topic-switching that occurred in the LDA of the reference data set compared to the joint model. Finally, we evaluated the similarity between the outputs of the matrix prior LDA and the joint model using Pearson's correlation, Spearman's correlation, and MSE. We focus on Pearson correlation in the main text but report other metrics in the Supplementary Material.

3. How is the number of topics K selected?

The number of topics was selected by using a hyperparameter search (Section 3.2.3) for the *C. elegans* data using a perplexity measure. We then multiplied the number of topics by 1.5, following (Durham et al., 2021). For the SHARE-seq mouse skin data, we found that the optimal number of topics was 2 according to the hyperparameter search; however, we suspected that this small value may arise because of the way that we subsampled the SHARE-seq data. Hence, we decided to instead use 15 topics to be consistent with the *C. elegans* data set. We also saw that, visually, we had more separation between previously called cell type labels using 15 topics in the SHARE-seq data. We have adjusted the wording in section 3.2.3 to provide a little more insight.

We found that the optimal number of topics was 10, that the perplexity was relatively insensitive to the choice of c_α, and that perplexity dropped with increasing values of c_B. Following Durham et al. (2021), we increased the number of topics to add some flexibility to the model, and set T to be 15 in our experiments. The results of the hyperparameter search and further comments are in Figures S1 and S2. UMAP plots of the results of LDA for different numbers of topics are shown in Figure S3.

We found that the optimal number of topics was 10, that the perplexity was relatively insensitive to the choice of c_α, and that perplexity dropped with increasing values of c_B. **Following Durham et al. (2021),**

which suggested that LDA models were robust to extra topics, we increased the number of topics to add some flexibility to the model, and set T to be 15 in our experiments. The results of the hyperparameter search and further comments are in Figures S1 and S2. UMAP plots of the results of LDA for different numbers of topics are shown in Figure S3.

4. In section 4.3, the authors implemented the method on SHARE-seq data, which has both scRNA-seq and scATAC-seq from the same cell. How did the authors deal with the different scale between scRNA-seq and scATAC-seq data?

Because the matrix prior is the topic-gene matrix from a uniform prior LDA applied to the reference data, the rows sum to 1, i.e. the gene probabilities for each topic sum to 1. Hence, the scale of the prior is normalized between the two data modalities. We have updated the text to make this point clear.

In addition to serving as another dataset to validate our scATAC-seq prior, the SHARE-seq co-assay data allowed us to evaluate whether a matrix prior generated from one data modality, scRNA-seq, could improve LDA performance on another data modality, scATAC-seq. Because the SHARE-seq scATAC-seq and scRNA-seq data were generated from the same cells, we were able to directly assess the agreement between the scRNA-seq LDA and the scATAC-seq LDA, with and without a matrix prior derived from the scRNA-seq data (Section 3.2.4). See Supplementary Note 1, where we report that the scRNA-seq prior was able to improve inference for moderate values of c_B but worsened inference for larger values.

In addition to serving as another dataset to validate our scATAC-seq prior, the SHARE-seq co-assay data allowed us to evaluate whether a matrix prior generated from one data modality, scRNA-seq, could improve LDA performance on another data modality, scATAC-seq. Because the SHARE-seq scATAC-seq and scRNA-seq data were generated from the same cells, we were able to directly assess the agreement between the scRNA-seq LDA and the scATAC-seq LDA, with and without a matrix prior derived from the scRNA-seq data (Section 3.2.4). See Supplementary Note 1, where we report that the scRNA-seq prior was able to improve inference for moderate values of c_B but worsened inference for larger values. **Note that although scRNA-seq and scATAC-seq produce data on different scales, this does not affect the matrix prior because it is based on the topic-gene probabilities (which sum to 1 for each topic) and not the counts**.

Reviewer 2

In this manuscript, the authors Min et al. developed a novel approach that leverage the information from the published large-scale single-cell genomic data to facilitate the analysis of new datasets. The approach could be very helpful for biologists working on single-cell epigenomic analysis, as it has been challenging to generate large-scale datasets due to the high cost of library preparation and sequencing. I am generally satisfied with the performance of the method and excited about its potential applications. Following are several comments that should be fixed before publication.

1. The authors should evaluate the performance of the technique for leveraging information from different datasets (e.g., generated by different scATAC-seq techniques or in different experiment batches).

We have already partially addressed this request in the case of scRNA-seq data (Supplementary Note 1), where we showed that when scRNA-seq data is used as a prior, we have some evidence that improvement is possible for moderate values of c_B. We believe that further analysis on different scATAC-seq techniques would be beyond the scope of the paper.

2. How does the data sparsity of scATAC-seq affect the performance of the method? It would be interesting to check whether the approach can be used to improve the clustering analysis of data with relatively shallow sequencing.

We agree that this is an interesting question, and we have performed some new experiments to address it. We approached data sparsity by downsampling the available data (both target and reference) and comparing the performance as a function of sparsity. Let X be the number of cut sites we observed that intersect with a gene body. For each such gene body, we then downsampled using varying binomial probabilities p to $\tilde{X} \sim Binomial(X, p)$, resulting in sparser data as p decreases (Figure R3). We then took the $p = 0.2$ and $p = 0.8$ data sets, and we conducted an analysis similar to Sections 3.2.3 and in response to reviewer 1,

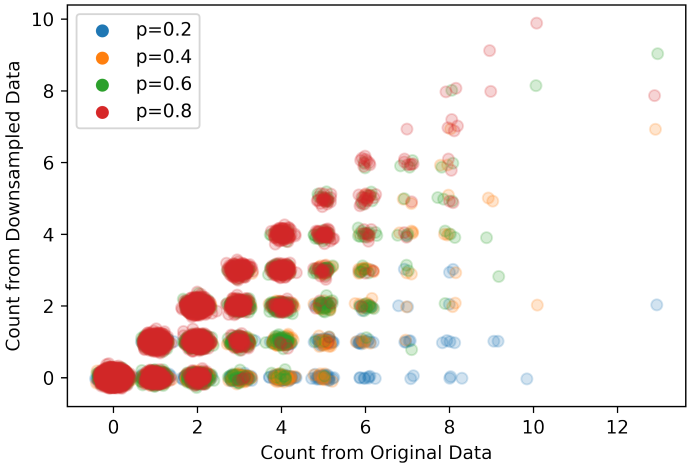

Figure R3: Different binomial probabilities (colors) were used to downsample both the target and reference data (Y-axis) and compared to the original data (X-axis). The entries from the first cell in the reference data are plotted. Random noise is added to both the original and downsampled data to aid in visibility.

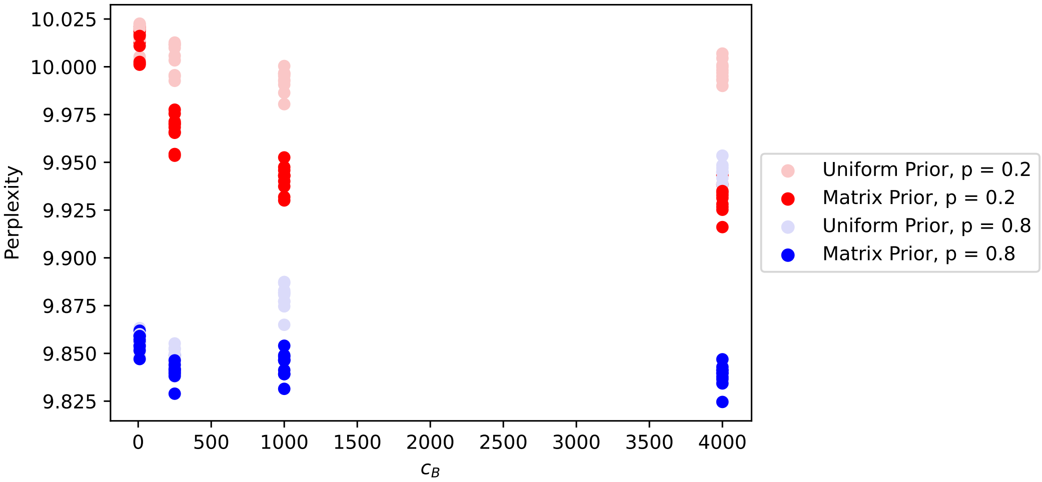

Figure R4: Perplexity scores (Y-axis) across different values of the concentration parameter c_B (X-axis) when data were downsampled with $p = 0.2$ (red) or $p = 0.8$ (blue), where each point represents a split of the target data set. We include both the uniform prior (lighter colors) and the matrix prior (darker colors)

comment 1. This analysis results in a perplexity score for each cell, which we aggregate into an average score for each split of the target data set (Figure R4). Lower perplexity is better. We compared the perplexity measure using both the matrix prior (darker colors) and the uniform prior (lighter colors). This was done with $p = 0.2$ and $p = 0.8$. We found that the matrix prior had lower perplexity than the uniform prior in both cases, indicating that the matrix prior is an improvement in both cases. We found that perplexity was higher when $p = 0.2$ than when $p = 0.8$, indicating a degradation of performance.

3. To facilitate the application of the method, it would be great if the authors could include an example (e.g., a test dataset, processing pipeline and analysis result) in the GitHub.

We have created a vignette that includes instructions on how to install the software, an example dataset, commands to run the software, and an analysis result. This is currently stored at https://github.com/Noble-Lab/lda_matrix_prior and is referenced in the manuscript in the Data Availability section.

Additional Changes

During the process of making revisions, we realized that we did not vary c_B for the uniform prior when comparing the matrix prior to the uniform prior (Figure 5). Some figures have been added to the revision to address this, which has changed some of the original figure numbers. When making this comparison, we found that the silhouette values using the matrix prior did not improve upon the uniform prior (Figure S16). We have hence also removed Figure S20. We have updated Figure 3 to Figure 4 to include a comparison of the uniform prior when c_B is increased, and we can still see the qualitative improvement of the clusters in the UMAP.

Because we still wanted a quantitative method demonstrating that the matrix prior improved upon the uniform prior, we compared the perplexity of the model on a held-out test set using the uniform prior versus the matrix prior, varying c_B on both (Figure 6). We found that the matrix prior improved on the perplexity measure.
